# Interleukin‐37 and Dendritic Cells Treated With Interleukin‐37 Plus Troponin I Ameliorate Cardiac Remodeling After Myocardial Infarction

**DOI:** 10.1161/JAHA.116.004406

**Published:** 2016-12-05

**Authors:** Ruirui Zhu, Haitao Sun, Kunwu Yu, Yucheng Zhong, Huairui Shi, Yuzhen Wei, Xin Su, Wenbin Xu, Quan Luo, Fangyuan Zhang, Zhengfeng Zhu, Kai Meng, Xiaoqi Zhao, Yuzhou Liu, Yi Mao, Peng Cheng, Xiaobo Mao, Qiutang Zeng

**Affiliations:** ^1^ Laboratory of Cardiovascular ImmunologyInstitute of CardiologyUnion HospitalTongji Medical CollegeHuazhong University of Science and TechnologyWuhanChina; ^2^ Department of CardiologyWuhan Union Hospital West CampusWuhanChina; ^3^ Department of DermatologyWuhan Union Hospital West CampusWuhanChina

**Keywords:** interleukin‐37, myocardial infarction, remodeling, tolerogenic dendritic cells, Treg cells, Myocardial Infarction, Remodeling

## Abstract

**Background:**

Excessive immune‐mediated inflammatory reactions play a deleterious role in postinfarction ventricular remodeling. Interleukin‐37 (IL‐37) emerges as an inhibitor of both innate and adaptive immunity. However, the exact role of IL‐37 and IL‐37 plus troponin I (TnI)–treated dendritic cells (DCs) in ventricular remodeling after myocardial infarction (MI) remains elusive.

**Methods and Results:**

MI was induced by permanent ligation of the left anterior descending artery. Our results showed that treatment with recombinant human IL‐37 significantly ameliorated ventricular remodeling after MI, as demonstrated by decreased infarct size, better cardiac function, lower mortality, restricted inflammatory responses, decreased myocardial fibrosis, and inhibited cardiomyocyte apoptosis. In vitro, we examined the phenotype of IL‐37 plus TnI–conditioned DCs of male C57BL/6 mice and their capacity to influence the number of regulatory T cells. Our results revealed that IL‐37 plus TnI–conditioned DCs obtained the characteristics of tolerogenic DCs (tDCs) and expanded the number of regulatory T cells when co‐cultured with splenic CD4^+^ T cells. Interestingly, we also found that adoptive transfer of these antigen‐loaded tDCs markedly increased the number of regulatory T cells in the spleen, attenuated the infiltration of inflammatory cells in the infarct hearts, decreased myocardial fibrosis, and improved cardiac function.

**Conclusions:**

Our results reveal a beneficial role of IL‐37 or tDCs treated with IL‐37 plus TnI in post‐MI remodeling that is possibly mediated by reestablishing a tolerogenic immune response, indicating that IL‐37 or adoptive transfer of IL‐37 plus TnI–treated tDCs may be a novel therapeutic strategy for ventricular remodeling after MI.

## Introduction

As a result of advances in aggressive revascularization and pharmacological therapy, mortality of acute myocardial infarction (MI) is significantly declining. However, ventricular remodeling, the process of complex architectural myocardial alteration after MI, is related to a poor clinical prognosis. Congestive heart failure (CHF) due to post‐MI ventricular remodeling is increasing and remains an unresolved problem worldwide. Therefore, it is important to elucidate the potential mechanisms involved in this process and search for alternative therapeutic targets against ventricular remodeling.

Inflammation and immune responses play a crucial role in the pathogenesis of post‐MI remodeling.^1^, ^2^, ^3^ It has been reported that toll‐like receptor 4 mediates maladaptive left ventricular (LV) remodeling and impairs cardiac function after MI.^4^ Moreover, experimental studies from our laboratory demonstrated that interleukin (IL)–17A promotes ventricular remodeling after MI.^5^ These findings suggest that excessive immune‐mediated inflammatory reactions play a deleterious role in postinfarction ventricular remodeling. Paradoxically, immunosuppressive therapy with methylprednisolone resulted in increased catastrophic mortality due to cardiac rupture.^6^, ^7^ Indeed, several experimental data show that macrophage colony‐stimulating factor (M‐CSF) treatment increases macrophage infiltration post‐MI, resulting in improved cardiac function and accelerated infarct repair, while macrophage depletion using clodronate‐containing liposomes impaired wound healing in a murine model.^8^, ^9^, ^10^ Therefore, controlled inflammation and immune response are prerequisites for adequate cardiac healing after MI. However, the exact mechanism that regulates these reactions in the post‐MI healing process remains to be elucidated.

IL‐37, formerly known as IL‐1F7, is expressed in peripheral blood mononuclear cells, dendritic cells (DCs), monocytes, and epithelial cells and acts as a natural inhibitor of innate and adaptive immunity.^11^, ^12^, ^13^ Low levels of steady‐state IL‐37 mRNA and protein are expressed physiologically.^14^ However, IL‐37 levels can be upregulated by stimulation with toll‐like receptor ligands or proinflammatory cytokines, which, in turn, inhibit the proinflammatory cytokines, such as IL‐1α, IL‐1β, IL‐6, granulocyte M‐CSF (GM‐CSF), and M‐CSF.^15^ Although the murine homolog of IL‐37 has yet to be found, human IL‐37 expression in a variety of murine cells suppresses innate and adaptive immunity and inhibits production of proinflammatory chemokines and cytokines, suggesting that human IL‐37 is still functional in murine cells.^12^, ^13^, ^16^, ^17^ Administration of human IL‐37 plasmid‐DNA in mice decreases local and systemic inflammation in psoriasis and concanavalin A–induced hepatitis, while IL‐37tg mice expressing human IL‐37b produce lower amounts of proinflammatory cytokines after lipopolysaccharide (LPS) treatment and are protected from dextran sulfate sodium–induced colitis and LPS‐induced septic shock compared with wild‐type mice.^12^, ^17^, ^18^, ^19^ More importantly, our previous study revealed that IL‐37 plays a regulatory role in myocardial ischemia/reperfusion injury by suppressing neutrophil infiltration and decreasing cardiomyocyte apoptosis.^20^ In spite of the previous study in mice providing some evidence for the beneficial role of IL‐37 in post‐MI remodeling,^21^ the underlying mechanisms in this process remain elusive.

DCs are professional antigen–presenting cells that orchestrate the defense against infectious agents, as well as the regulation of peripheral tolerance.^22^, ^23^ Although mature DCs (mDCs) play an immunogenic role in the development of effector T cells, semimature tolerogenic DCs (tDCs) can induce and maintain peripheral T‐cell tolerance.^24^ It has been reported that tDCs produce more IL‐10 and indolamine 2, 3‐dioxygenase (IDO) than mDCs and have impaired abilities to synthesize IL‐12p70.^25^ Treatment of DCs with the immunosuppressive cytokine IL‐10 decreases secretion of inflammatory cytokines such as tumor necrosis factor‐α (TNF‐α), IL‐6, and IL‐12 and promotes generation of regulatory T cells (Tregs).^26^, ^27^ Furthermore, accumulating evidence indicates that specific antigen‐loaded tDCs can suppress the onset and/or progression of autoimmune disease in several models, such as experimental autoimmune encephalitis, collagen‐induced arthritis, or experimental autoimmune myocarditis.^28^, ^29^, ^30^ However, the utility of these immunoregulatory DCs as treatment for post‐MI remodeling has not been elucidated.

The primary aim of the present study was to clarify the effects of IL‐37 and DCs treated with troponin I (TnI) and IL‐37 in post‐MI remodeling. We found that IL‐37 plus TnI imposes a regulatory phenotype on DCs, and that such tDCs promote induction of Tregs. We have further shown that intraperitoneal injections of IL‐37 or a single intravenous injection of IL‐37 and TnI‐induced tDCs alleviates cardiac remodeling after MI. Our data are the first to report that IL‐37 plus TnI–treated DCs induce a tolerogenic immune response and ameliorate post‐MI remodeling in mice.

## Materials and Methods

### Animals

Male C57BL/6 mice aged 6 to 10 weeks were purchased from Beijing HFK Bioscience Co., Ltd (Beijing, China). The mice were maintained on a chow diet in the Tongji Medical College Animal Care Facility according to institutional guidelines. All animal studies were approved by the Animal Care and Utilization Committee of Huazhong University of Science and Technology. Institutional review board approval was also obtained.

### Cell Culture

Bone marrow–derived DCs (BMDCs) were generated with GM‐CSF and IL‐4 as previously described.^31^ In brief, bone marrow was isolated from 6‐week‐old male C57BL/6 mice. Bone marrow cells were depleted of red blood cells and were cultured in RPMI 1640 supplemented with 10% FCS (GIBCO, Carlsbad, CA), 100 U/mL penicillin, 100 U/mL streptomycin, 20 ng/mL recombinant mouse GM‐CSF (Peprotech, Rocky Hill, NJ), and 10 ng/mL IL‐4 (Peprotech) at 37°C with 5.0% CO_2_. On day 3, nonadherent cells were washed and re‐fed the same concentrations of GM‐CSF and IL‐4. On day 6, half of the culture medium was replaced with fresh medium using the same culture conditions. After 8 days of culture, immature DCs (imDCs) were obtained. Purification of DCs from the differentiated bone marrow cells was performed with a CD11c magnetic cell‐sorting kit (Miltenyi Biotec, Auburn, CA) according to the manufacturer's instructions. The detailed methods to generate different DC subsets are shown in Table S1. Unless otherwise indicated, DCs treated with IL‐37 plus TnI should be referred to as tDCs.

Neonatal cardiomyocytes were isolated and cultured using previously described methods, with some modifications.^32^ Briefly, the hearts from 1‐day‐old C57BL/6 mice were removed after cervical dislocation, cut into small chunks, and washed with Hanks' balanced salt solution. Then, the tissue was incubated in 4 mL trypsin/EDTA solution (GIBCO, Carlsbad, CA) at 4°C for 30 minutes with rotation. The digestion was stopped by addition of 6 mL DMEM containing 20% FCS (GIBCO). After centrifugation at 150g for 5 minutes, the supernatant was removed and the tissues were incubated in 4 mL Liberase TH (0.1 U/mL in Hanks' balanced salt solution, Roche Diagnostics GmbH, Mannheim, Germany) at 37°C for 15 minutes. The supernatant containing the released cells to DMEM‐20% FCS was removed and fresh Liberase TH was added to the undigested tissues, which were then incubated for a further 15 minutes. This digestion procedure was repeated until most of the cells had been released from ventricular tissue and the obtained cells were resuspended in DMEM. All collected cells were filtered through a nylon cell strainer (70 μm size; BD Falcon, Franklin Lakes, NJ) and seeded into fibronectin‐coated 12‐well tissue culture plates (Costar, Corning, NY). After 1 hour of incubation with 5% CO_2_ at 37°C, the attached fibroblasts were discarded and cardiomyocytes in the supernatant were enriched and seeded into fibronectin‐coated tissue culture plates after cell concentration was adjusted. Cardiomyocytes were used in experiments when they had formed a confluent monolayer and beat in synchrony at 72 hours. Cultured cardiomyocytes were exposed to H_2_O_2_ (200 μmol/L) (Sigma‐Aldrich, St. Louis, MO) and/or recombinant human IL‐37 (30 ng/mL) (Adipogen AG, Liestal, Switzerland), as indicated, for 4 hours and expression of Bax and Bcl‐2 mRNA levels was measured by using the real‐time polymerase chain reaction (RT‐PCR) protocol.

### DC/T‐Cell Co‐Culture

imDCs, tDCs, or mDCs (2×10^5^ cells/mL) were co‐cultured with CD4^+^ T cells (isolated using a CD4 MicroBead mouse kit: Miltenyi Biotec) (1×10^6^ cells/mL) from splenocytes of male C57BL/6 mice. The mixed cells were cultured for 3 days at 37°C with 5.0% CO_2_ in 2 mL RPMI 1640 supplemented with 10% FCS. For Treg cells analysis by flow cytometry, the mixed cells were stained with anti‐CD4‐FITC, anti‐CD25‐APC, and then anti‐Foxp3‐PE (eBioscience, San Diego, CA) according to the manufacturer's instructions.

### MI Model and Assessment of Infarct Size and Fibrosis

MI was induced by permanent ligation of the left anterior descending (LAD) artery as previously described.^33^ In brief, mice were anesthetized with an intraperitoneal injection of ketamine (80 mg/kg) and xylazine (10 mg/kg), and, after the initiation of anesthesia, the mice were intubated and ventilated by a rodent ventilator. A small skin cut (1.2 cm) was made over the left chest, and then the fourth intercostal space was exposed after dissection and retraction of the pectoral major and minor muscle. To open the pleural membrane and pericardium, a small hole was made at the fourth intercostal space by a mosquito clamp. With the clamp slightly open, the heart was smoothly “popped out” through the hole. After location, the LAD was sutured and ligated at a site 3 mm from its origin using a 6‐0 silk suture. Complete occlusion of the LAD was deemed successful when the anterior wall of the left ventricle turned pale and the ST segment was elevated (Figure S1). The heart was then immediately placed back into the intrathoracic space followed by manual evacuation of air and closure of muscle and the skin. The sham group underwent the same surgical procedure except that the LAD was not ligated. Mice were sacrificed at the designated times.

The infarct area after 1 day was determined by 2, 3, 5‐triphenyltetrazolium chloride (TTC) staining.^34^ Briefly, 1 day post‐MI, frozen hearts were acquired and cut into 5 sections from the apex to the base. Sections were then incubated in TTC (Sigma‐Aldrich) phosphate buffered solution (pH 7.4) at 37°C for 15 minutes. The viable tissue was stained red, whereas the infarcted tissue was white. The extent of fibrosis was measured using Masson's trichrome (Good Bioscience Co., Ltd, Wuhan, China) on day 28 after MI.^35^ Image‐Pro Plus version 6.0 software (Media Cybernetics, Inc., Bethesda, MD) was used to determine the infarct size and extent of fibrosis.

### Treatment and Groups

C57BL/6 mice underwent sham surgery or LAD ligation and were analyzed on days 1, 3, 7, 14, and 28 after MI. To investigate the role of IL‐37 in post‐MI remodeling, C57BL/6 mice were randomly assigned to 1 of 3 groups and were analyzed at the designated times. The groups were as follows: (1) the sham group (sham), in which C57BL/6 mice were subjected to sham operation; (2) the recombinant human IL‐37 (Adipogen AG, Liestal, Switzerland) treatment group (IL‐37+MI); and (3) the PBS treatment group (PBS+MI), in which the mice were injected intraperitoneally with 1 μg of recombinant human IL‐37 diluted in 200 μL PBS or with 200 μL PBS only, respectively, 15 minutes prior to MI or 24 hours after MI. In addition to the above treatment, 1 μg of recombinant human IL‐37 diluted in 200 μL PBS or 200 μL PBS was injected intraperitoneally twice per week until the designated time point after surgery.

To explore the effect of IL‐37 plus TnI–treated tDCs in ventricular remodeling after MI, adoptive transfer experiments of DCs were performed. C57BL/6 mice were randomly assigned to one of the following 4 groups and sacrificed at the designated times. These C57BL/6 mice underwent LAD ligation and received one intravenous injection of imDCs (the imDCs group), tDCs (the tDCs group), mDCs (the mDCs group) (1×10^6^ cells/mouse), or PBS alone (the no DCs group).

### Echocardiography

Echocardiography was performed using a Vevo 1100 high‐resolution microimaging system with a 30 MHz transducer (Visualsonic, Canada). Mice were anesthetized with 1.5% isoflurane, and 2‐dimensional mode and M‐mode images were recorded to assess cardiac function. LV end‐diastolic diameter (LVEDD), LV end‐systolic diameter (LVESD), ejection fraction (EF), and fractional shortening (FS) were calculated on days 7 and 28 after MI as previously described.^33^

### Immunohistochemistry Analysis

Immunohistochemical studies were performed by immunoperoxidase methods using paraffin‐embedded tissue sections (6 mm thick). The sections were stained with haematoxylin and eosin to identify infarct area. The sections were incubated with primary anti‐myeloperoxidase (MPO; Abbiotec, UK), anti‐Mac3 (BD Bioscience), and anti‐CD3 (eBioscience) at 4°C overnight, followed by respective secondary HRP‐conjugated antibodies for 1 hour at room temperature. The positive cells were visualized with DAB, and nuclei were counterstained with hematoxylin. The numbers of MPO^+^ neutrophils, mouse CD107b (Mac3^+^) macrophages, and CD3^+^ T lymphocytes were assessed by counting the total cell numbers in the infarcted and border areas in 10 randomly chosen fields in each section.

### TUNEL Staining

The paraffin‐embedded heart sections were stained using the In Situ Cell Death Detection kit (Roche Diagnostics GmbH, Mannheim, Germany) according to the manufacturer's instructions. Next, the sections were costained with an anti‐actinin antibody (Sigma‐Aldrich) to specifically label the cardiomyocytes. Tetramethylrhodamine goat anti‐mouse antibody was used as the secondary antibody. Cell nuclei were counterstained with 4, 6‐diamidino‐2‐phenylindole. More than 5 fields in >3 different sections/animals were examined in a blinded fashion by a technician who was not informed about the treatment groups.

### RT‐PCR Analysis

Total RNA was extracted from tissues or cells using Trizol (Takara Biotechnology, Dalian, China) and reverse transcribed into cDNA using the PrimeScript RT reagent kit (Takara Biotechnology, Dalian, China) according to the manufacturer's instructions. The mRNA levels of the target genes were quantified using SYBR Green Master Mix (Takara Biotechnology) with an ABI PRISM 7900 Sequence Detector system (Applied Biosystems, Foster City, CA). Each reaction was performed in triplicate, and changes in the relative gene expression level normalized to the GAPDH level were calculated using the relative threshold cycle method. The primer sequences are shown in Table.

**Table 1:** Primers Used for RT‐PCR

Gene	Forward (5′‐3′)	Reverse (5′‐3′)
IL‐6	CCGGAGAGGAGACTTCACAG	TCCACGATTTCCCAGAGAAC
IL‐1β	CTGTGACTCGTGGGATGATG	GGGATTTTGTCGTTGCTTGT
TNF‐α	TGCCTCAGCCTCTTCTCATT	GCTTGGTGGTTTGCTACGAC
IL‐10	GCTCTTACTGACTGGCATGAG	CGCAGCTCTAGGAGCATGTG
TGF‐β	TGCTTCAGCTCCACAGAGAA	TGGTTGTAGAGGGCAAGGAC
Bax	TGCAGAGGATGATTGCTGAC	GATCAGCTCGGGCACTTTAG
Bcl‐2	GTACCTGAACCGGCATCTG	GCTGAGCAGGGTCTTCAGAG
IDO	CAGCTTCTCCTGCAATCAAAGCA	TGCGAGGTGGAACTTTCTCACAGA
IFN‐γ	ATGAACGCTACACACTGCATC	CCATCCTTTTGCCAGTTCCTC
IL‐12	ATCGTTTTGCTGGTGTCTCC	CTTTGTGGCAGGTGTACTGG
Foxp3	TCAAAGAGCCCTCACAACCAGCTA	TTTGAAGGTTCCAGTGCTGTTGC
IL‐17A	TGTGAAGGTCAACCTCAAAGTCT	GAGGGATATCTATCAGGGTCTTCAT
GAPDH	GTGCTGAGTATGTCGTGGAG	GTCTTCTGAGTGGCAGTGAT

All of these primers were synthesized by Tsingke in Wuhan. IDO indicates indolamine 2, 3‐dioxygenase; IFN‐γ, interferon‐γ; IL, interleukin; RT‐PCR, real‐time polymerase chain reaction; TGF‐β, transforming growth factor‐β; TNF‐α, tumor necrosis factor‐α.

### Western Blot Analysis

Protein extracted from heart tissues was separated on 10% SDS‐PAGE and transferred to polyvinylidene difluoride membranes. After being blocked with 5% defatted milk, the membranes were incubated with the appropriate primary antibodies at 4°C overnight, followed by incubation with an HRP‐conjugated secondary antibody. The antibodies to Matrix metalloproteinase (MMP) 2, IL‐6, IL‐1β, and TNF‐α were from Santa Cruz Biotechnology, and the anti‐TGF‐β, anti‐IL‐10, and anti‐GAPDH antibodies were from R&D System. The specific bands were detected using the Super ECL reagent (Thermo Scientific). Images were obtained and analyzed with Image Lab 3.0 software (Bio‐Rad Laboratories, Hercules, CA).

### Flow Cytometry

One week after MI, the mononuclear cells from the spleen were isolated with Ficoll‐Paque PLUS (MP Biomedicals), and the erythrocytes were removed by RCLB. For detection of CD4^+^CD25^+^Foxp3^+^ Treg cells, the cells were stained with anti‐CD4‐FITC and anti‐CD25‐APC and then stained with anti‐Foxp3‐PE after fixation and permeabilization according to the manufacturer's instructions. For analysis of T helper (Th)1 (CD4^+^interferon‐γ [IFN‐γ^+^]) and Th17 (CD4^+^IL‐17^+^), the mononuclear cells were suspended at a density of 2×10^6^ cells/mL in RPMI 1640 supplemented with 10% FCS (GIBCO). The cell suspension in 1 mL of medium was transferred to each well of 24‐well plates. Cultures were stimulated with phorbol myristate acetate (20 ng/mL) plus ionomycin (1 μg/mL) (Alexis Biochemicals) for 4 hours in the presence of 2 μmol/mL monensin (Alexis Biochemicals). The incubator was set at 37°C under a 5% CO_2_ environment. After 4 hours of culture, the mononuclear cells were collected for staining according to the instructions. Fixation and permeabilization were necessary before staining with IFN‐γ or IL‐17 antibody. Isotype controls were given to enable correct compensation and confirm antibody specificity. For analysis of the characterization for cultured DCs, the purified CD11c^+^ cells were stained with MHC‐II‐FITC, CD40‐PE, or CD86‐PE for 30 minutes. All of the antibodies for flow cytometry were from eBioscience (San Diego, CA). Flow cytometric acquisition was performed using FACSCalibur (BD Immunocytometry Systems), and all analyses were performed using FlowJo software (FlowJo, LLC, Ashland, OR).

### Statistical Analysis

Data are presented as mean±SEM. Differences were evaluated using unpaired Student *t* test between 2 groups and 1‐way ANOVA for multiple comparisons, followed by a post hoc Student‐Newmann‐Keuls test when necessary. Two‐factor ANOVA was used to make comparisons over time by groups. Survival was analyzed by the method of Kaplan and Meier with statistical differences between the mortality curves assessed using log‐rank test. All analyses were done using GraphPad Prism 6.0 (GraphPad Software, Inc, La Jolla, CA), and statistical significance was set at *P*<0.05.

## Results

### The Role of IL‐37 on Survival, Infarct Size, and Cardiac Function

**Figure 1:**
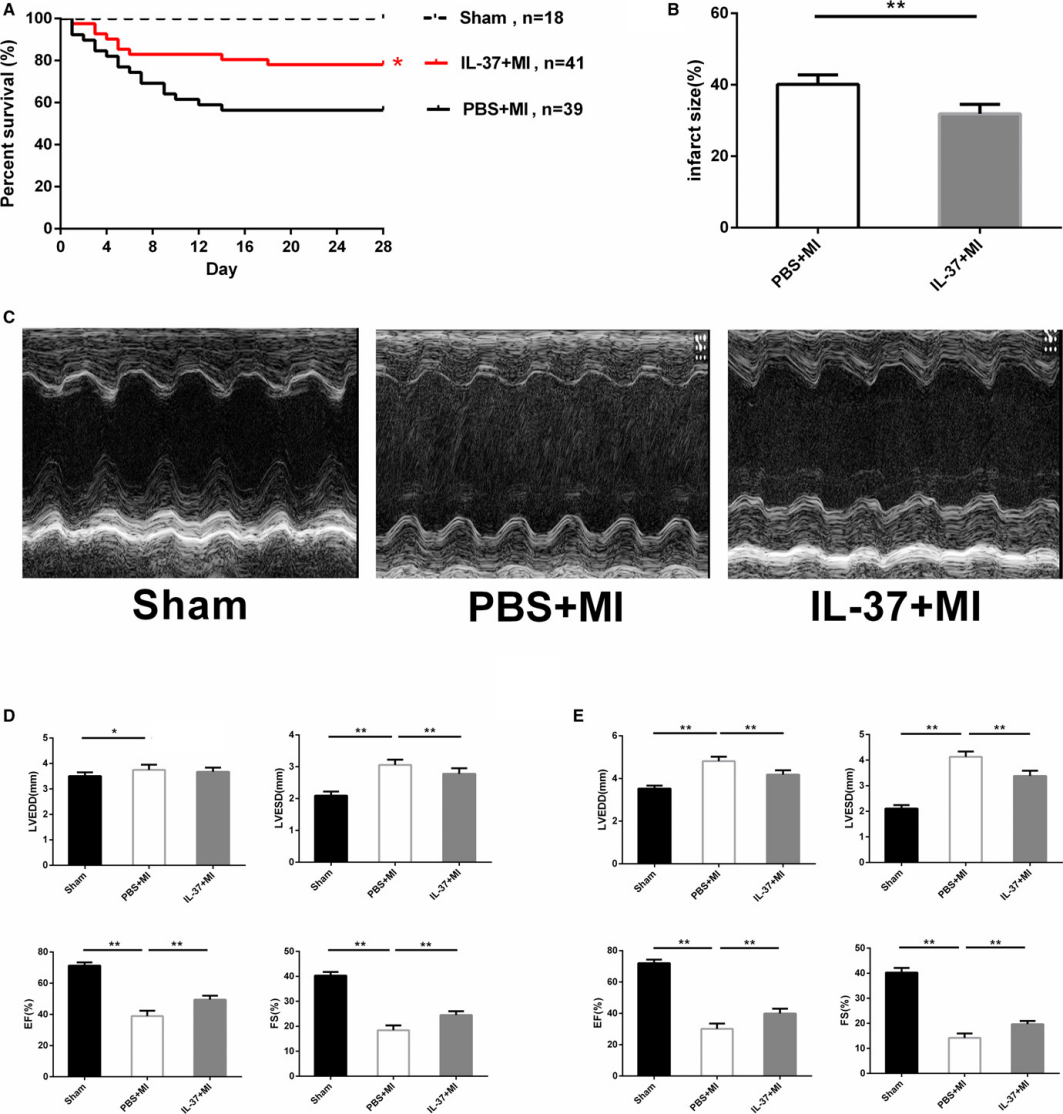
Interleukin (IL)–37 reduces myocardial infarct size and improves left ventricular function following myocardial infarction (MI). A, Survival analysis in PBS‐treated and IL‐37–treated mice after MI. B, Infarct size determined by 2, 3, 5‐triphenyltetrazolium chloride staining of sections on day 1 post‐MI (PBS‐treated group n=6 and IL‐37–treated group n=8). C, Representative M‐mode echocardiography images of the left ventricle on day 28 post‐MI. Left ventricular end‐diastolic dimension (LVEDD), left ventricular end‐systolic dimension (LVESD), ejection fraction (EF%), and fractional shortening (FS%) on day 7 (D) and day 28 (E) post‐MI (Sham n=6, PBS‐treated group n=6, and IL‐37‐treated group n=8). *P<0.05 and **P<0.01.

To determine the function of IL‐37 in MI mice, recombinant human IL‐37 was used. The survival rate on day 28 post‐MI was 56.41% (22/39) in PBS‐treated mice and 78.05% (32/41) in IL‐37–treated mice, indicating that IL‐37 decreased post‐MI mortality (Figure 1A). To investigate the role of IL‐37 in early‐phase ventricular remodeling, we measured the infarct size by TTC staining on day 1 after MI. The infarct size was significantly lower in the IL‐37–treated group and higher in the PBS‐treated group on day 1 post‐MI (Figure 1B).

Cardiac dysfunction and ventricular dilation happened 1 week post‐MI, altered rapidly at 2 weeks, and then changed slowly from 1 month.^36^ Therefore, we calculated LVEDD, LVESD, EF, and FS by echocardiography both on days 7 and 28 post‐MI. Compared with PBS‐treated mice, echocardiographic assessment consistently revealed that EF and FS were greater both on days 7 and 28 post‐MI, and LVEDD was smaller on day 28 after MI in IL‐37–treated mice (Figure 1C through 1E). Thus, cardiac function was significantly prevented in the IL‐37–treated group.

### Decreased Cardiac Fibrosis in IL‐37–Treated Hearts After MI

**Figure 2:**
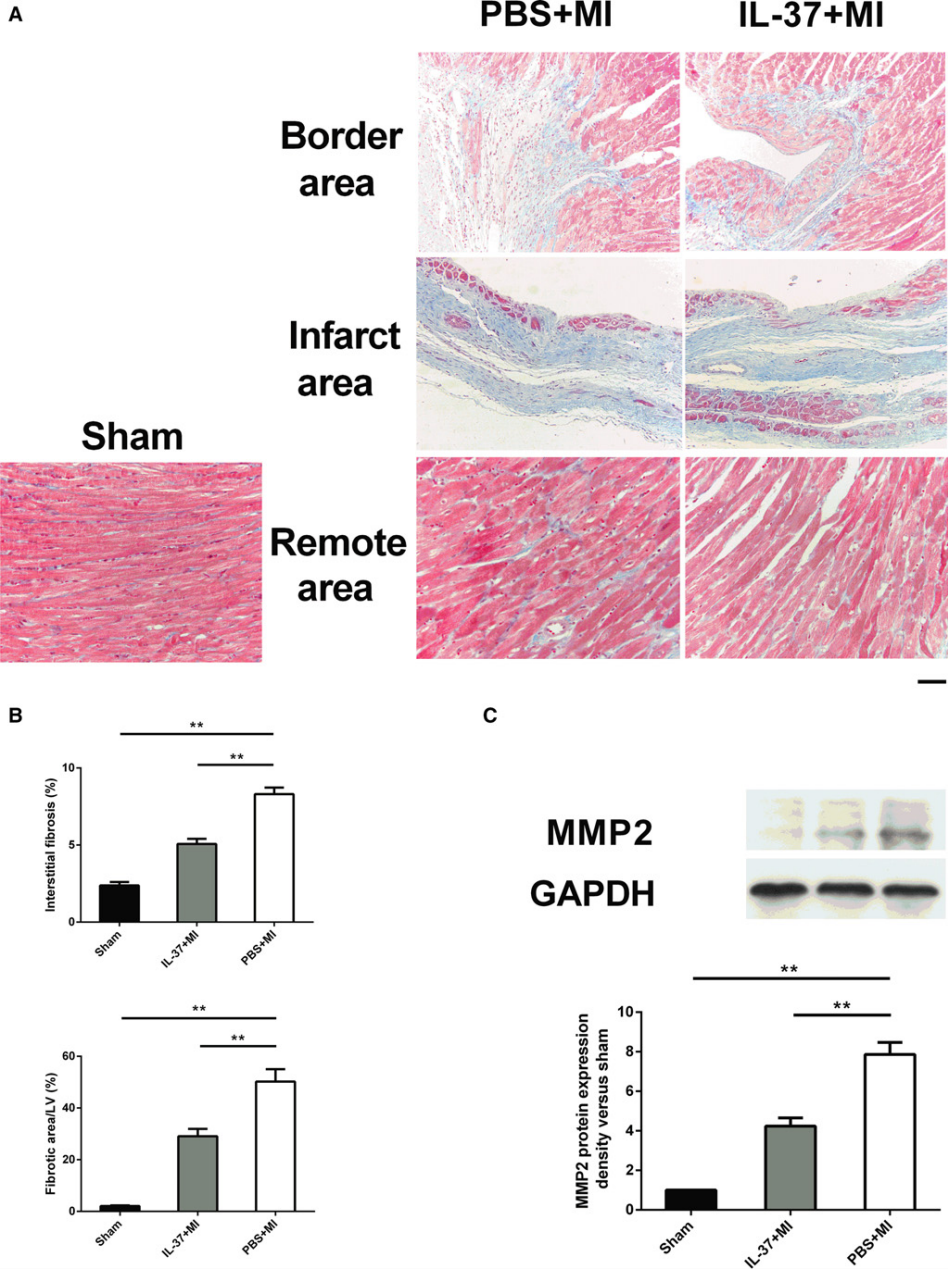
Changes in fibrosis and matrix metalloproteinase 2 (MMP2) expression after myocardial infarction (MI) in experimental groups. A, Representative Masson's trichrome staining images of collagen deposition (blue) in both infarct and remote areas on day 28 post‐MI. B, The extent of fibrosis, as assessed by the fibrotic area/left ventricle and interstitial fibrosis, was compared among the different groups (n=6 each). C, Representative images of Western blot demonstrating MMP2 expression in heart tissues on day 7 post‐MI (upper) and the data are presented as the fold change vs the sham group (lower) (n=6 each). Scale bar: 100 μm. **P<0.01.

Cardiac fibrosis is a critical character of post‐MI ventricular remodeling. Therefore, we measured fibrosis as the fibrotic area in infarct area and interstitial fibrosis in remote area. As shown in Figure 2A and 2B, IL‐37–treated mice exhibited markedly reduced fibrotic areas in both infarct and remote areas. MMPs play an important part in the extracellular matrix balance and ventricular remodeling post‐MI.^37^ Results from Western blot analysis showed that the expression of MMP2 was significantly lower in IL‐37–treated mice than that in controls (Figure 2C).

### IL‐37 Inhibits Inflammatory Response in the Infarcted Heart

**Figure 3:**
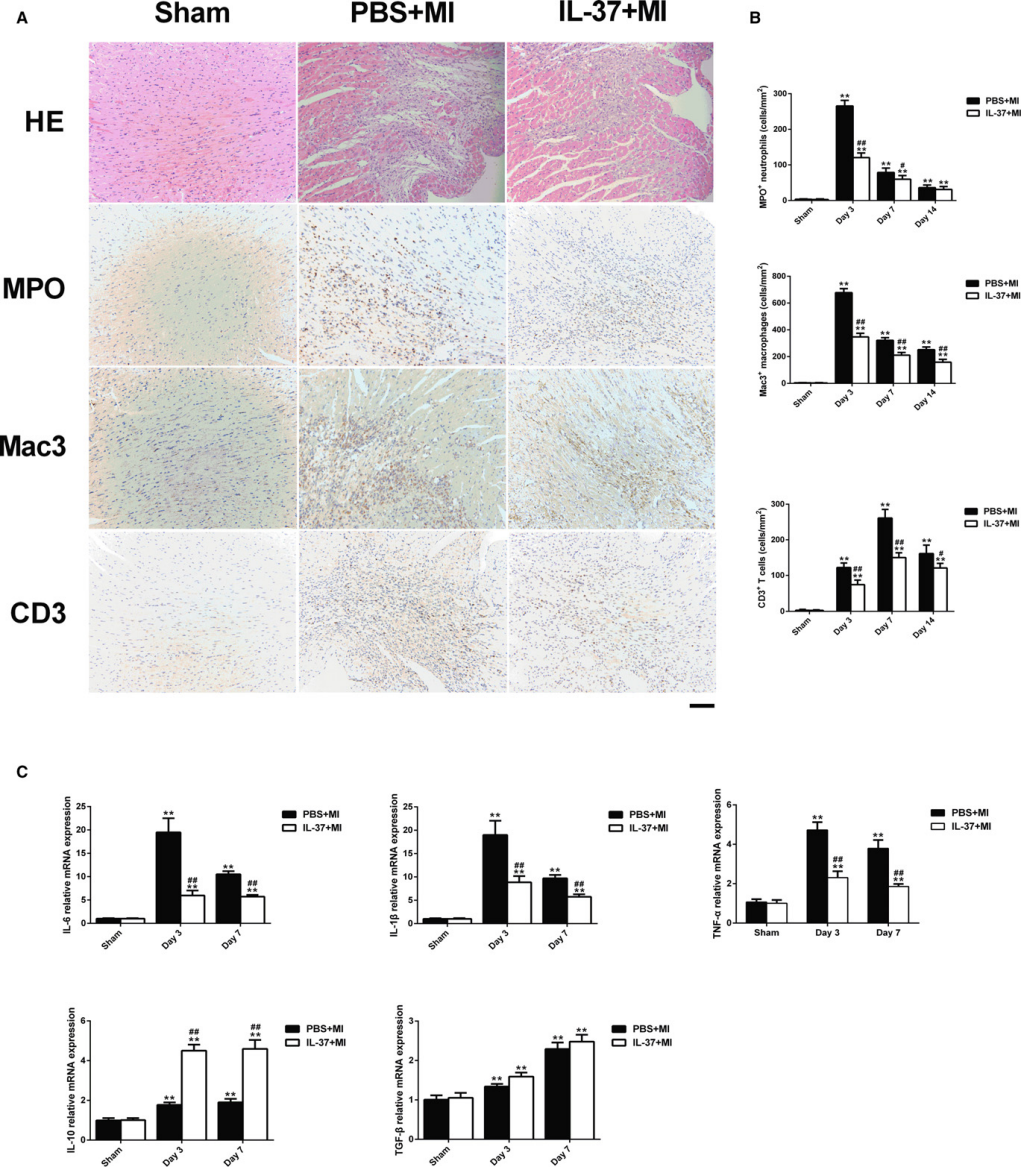
Inflammatory cells infiltration and cytokines expression in the infarcted heart. A, Representative images of haematoxylin and eosin (HE) staining, infiltration of myeloperoxidase (MPO^+^) neutrophils, mouse CD107b (Mac3^+^) macrophages, and CD3^+^ T cells in the border area of the infarct hearts. Images for neutrophils and macrophages are from day 3 after myocardial infarction (MI), and images for T cells are from day 7 post‐MI. B, Infiltration of neutrophils, macrophages, and T cells were compared between the different groups at set time points. C, Analysis of mRNA levels of proinflammatory cytokines interleukin (IL)–6, IL‐1β, and tumor necrosis factor‐α (TNF‐α), and anti‐inflammatory cytokines IL‐10 and transforming growth factor‐β (TGF‐β) on days 3 and 7 after MI. Data are depicted as fold changes vs sham. n=5 per group. Scale bar: 100 μm. **P<0.01 vs sham and ^#^ *P*<0.05, ^##^ *P*<0.01 vs PBS+MI.

Because of the anti‐inflammatory character of IL‐37, we investigated whether the beneficial effects of IL‐37 on ventricular remodeling are associated with the suppressed inflammatory responses. We first evaluated the infiltration of various inflammatory cells after MI in the infarcted hearts. The temporal pattern of the infiltration differed among inflammatory cells (Figure 3A and 3B). Results from immunohistochemical staining for MPO showed that the number of neutrophils that infiltrated into the infarcted myocardium peaked on day 3 after MI and was significantly lower in IL‐37–treated mice 3 and 7 days after MI than in control mice (Figure 3B). Macrophage infiltration revealed a similar temporal pattern, and the number of Mac3^+^ macrophages in the infarcted heart tended to be markedly decreased in IL‐37+MI group compared to PBS+MI group at all tested time points (Figure 3B). In addition, the infiltration of T lymphocytes reached a maximum on day 7, and then gradually decreased. The number of CD3 T cells that infiltrated the infarcted myocardium was downregulated in IL‐37–treated mice at all tested time points (Figure 3B).

Inflammatory cytokines play a central role in the progression of ventricular remodeling.^2^ We thus investigated the role of IL‐37 on the levels of local inflammatory factors. IL‐37 significantly reduced the mRNA levels of proinflammatory cytokines IL‐6, IL‐1β, and TNF‐α. On the other hand, the mRNA expression of IL‐10, which is anti‐inflammatory, was marked upregulated by IL‐37. However, the expression of TGF‐β was not affected (Figure 3C). Findings from Western blotting analysis further confirmed that the protein levels of these cytokines were similar with mRNA levels (Figure S2).

### IL‐37 Inhibits Cardiomyocyte Apoptosis In Vivo and In Vitro

**Figure 4:**
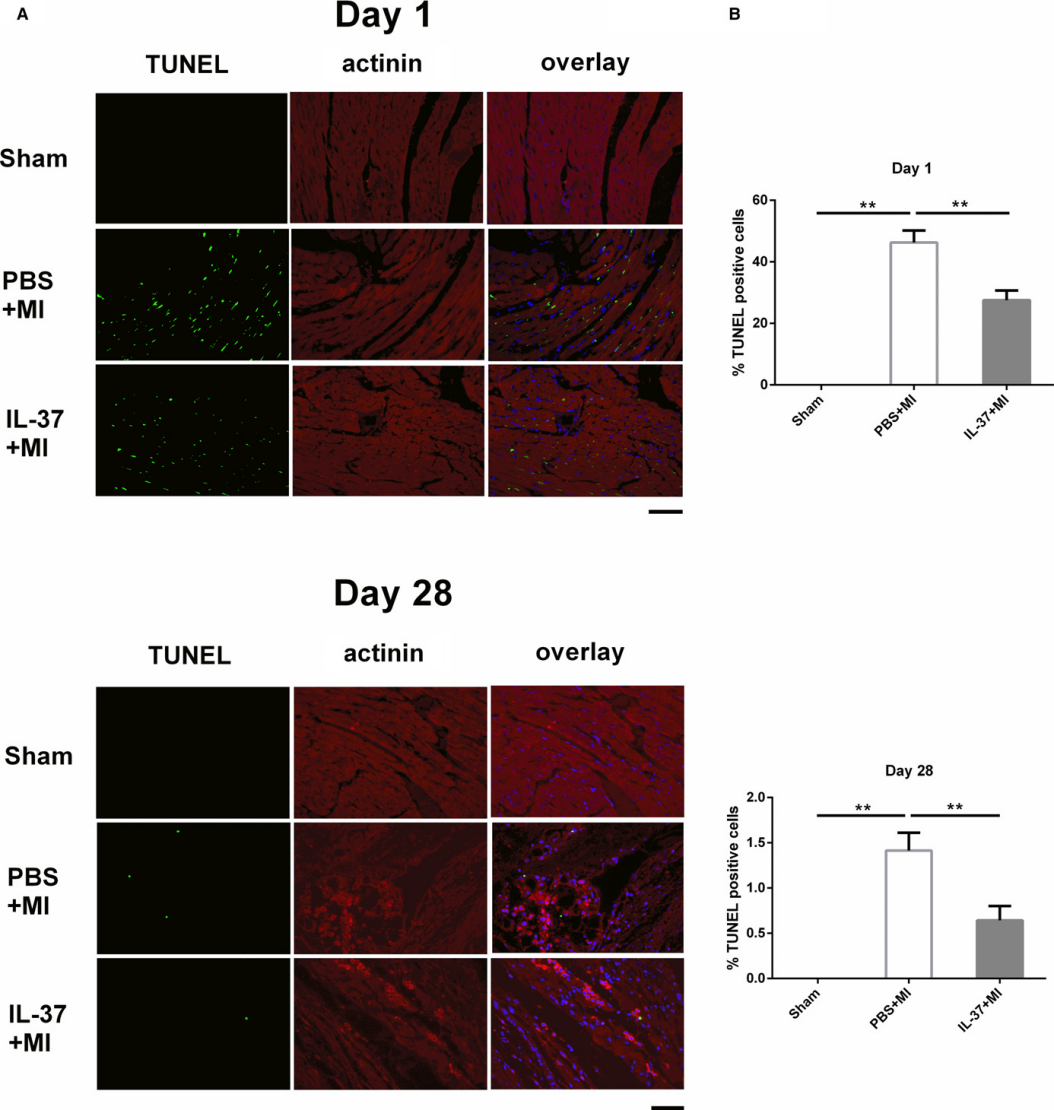
Interleukin (IL)–37 inhibited cardiomyocyte apoptosis in vivo. A, Representative images of terminal deoxynucleotidyl transferase dUTP nick‐end labeling (TUNEL)–stained heart sections from different groups 1 day and 28 days post–myocardial infarction (MI). TUNEL (green) and 4, 6‐diamidino‐2‐phenylindole (blue) staining of nuclei in apoptotic cardiomyocytes (red) in the peri‐infarct zone. B, Quantitative analysis of the percentages of TUNEL‐positive nuclei (n=6). Scale bar: 100 μm. **P<0.01.

To determine the mechanism underlying how IL‐37 prevents the development of LV remodeling, we first measured the number of apoptotic cells by terminal deoxynucleotidyl transferase dUTP nick‐end labeling (TUNEL) staining on days 1 and 28 after MI. As shown in Figure 4, the number of TUNEL‐positive cells was significantly smaller in IL‐37–treated hearts than in controls, indicating that IL‐37 has an antiapoptotic effect on cardiomyocytes in vivo in both early and late stages post‐MI. RT‐PCR analysis revealed that the expression of proapoptotic molecules Bax was less in IL‐37–treated mice than in controls. In contrast, the expression of antiapoptotic molecule Bcl‐2 was much greater in IL‐37–treated mice than in controls (Figure S3A). In addition, the Bax/Bcl‐2 ratio was significantly increased in cardiomyocytes exposed to H_2_O_2_, whereas when it was supplemented with IL‐37 it decreased the ratio (Figure S3B). Taken together, these data show that IL‐37 inhibits cardiomyocyte apoptosis, at least in part, through downregulation of proapoptotic to antiapoptotic molecule ratio of Bcl‐2 family.

### IL‐37 Expands Treg Cells but Suppresses Th1 and Th17 Cells In Vivo

**Figure 5:**
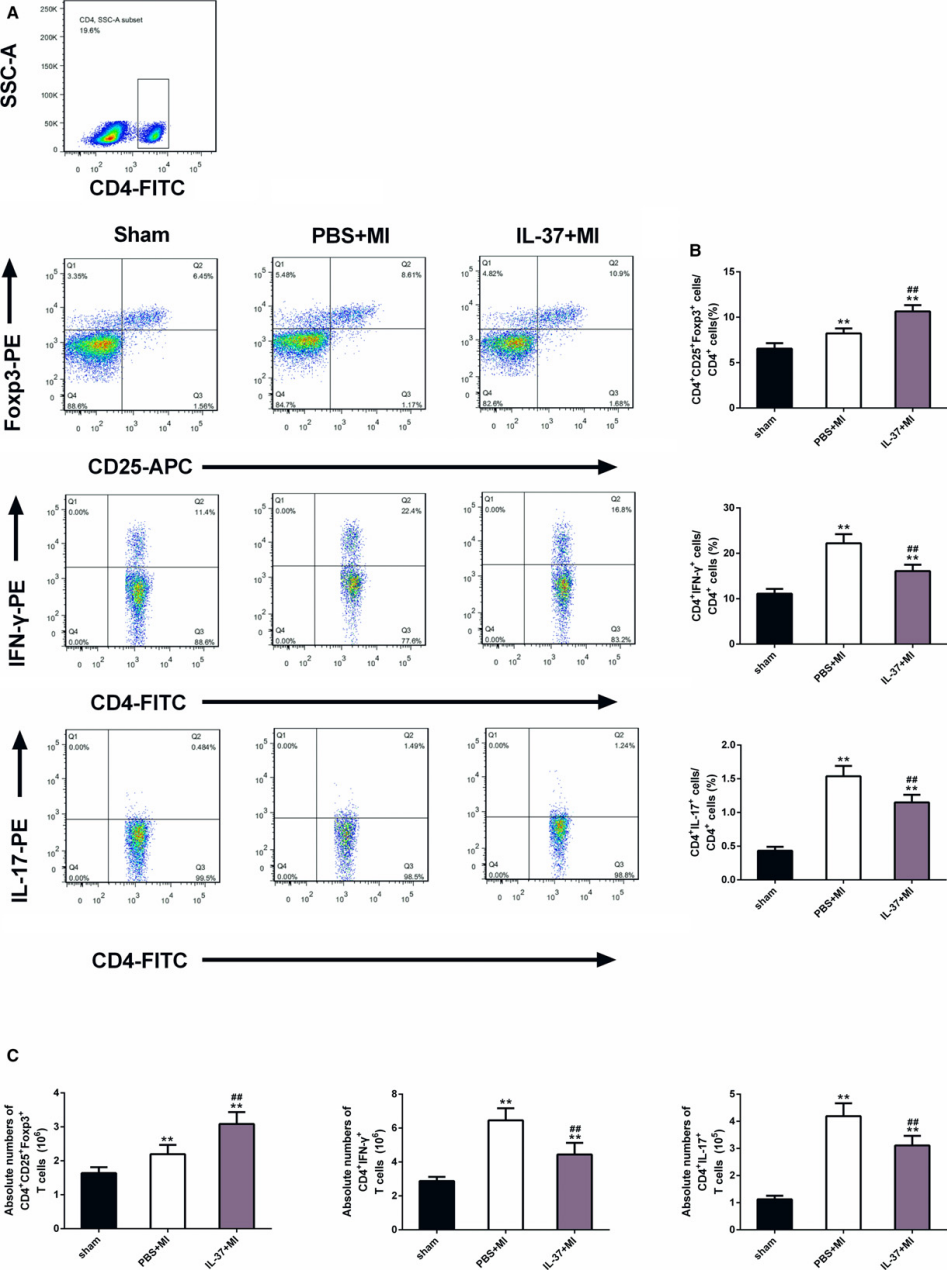
Effects of interleukin (IL)–37 on regulatory T cells (Tregs), T helper (Th)1, and Th17 cells in spleens of C57BL/6 mice on day 7 after myocardial infarction (MI). A, CD4^+^ T‐cell subsets were gated, and representative images of Tregs, Th1, and Th17 cells are shown. B, Results of statistical analysis of average percentages of Tregs, Th1, and Th17 cells. C, Absolute numbers of Tregs, Th1, and Th17 cells were counted in the spleen. n=6 per group. APC indicates activated protein C; FITC, fluorescein isothiocyanate. **P<0.01 vs sham and ^##^ *P*<0.01 vs PBS+MI.

We next investigated whether the beneficial role of IL‐37 in post‐MI remodeling was associated with the induction of anti‐inflammatory Treg cells and inhibition of proinflammatory Th1 and Th17 cells. CD4^+^ T‐cell subtypes in the spleen 7 days after MI were examined by flow cytometric analysis. We found that the proportion of Tregs, Th1, and Th17 cells was increased by permanent coronary occlusion compared with sham mice. IL‐37 markedly increased the percentage of CD4^+^CD25^+^Foxp3^+^ Tregs, whereas the number of CD4^+^IFN‐γ^+^ T cells and CD4^+^IL‐17^+^ T cells was significantly decreased in IL‐37–treated mice than in controls (Figure 5A and 5B). Similar results were found for absolute numbers of CD4^+^CD25^+^Foxp3^+^ Tregs, CD4^+^IFN‐γ^+^ T cells, and CD4^+^IL‐17^+^ T cells (Figure 5C). These results indicate that IL‐37 promotes the pool of splenic Tregs and suppresses the number of Th1 and Th17 cells in post‐MI mice.

### DCs Treated With IL‐37 and TnI Display a Tolerogenic Phenotype

**Figure 6:**
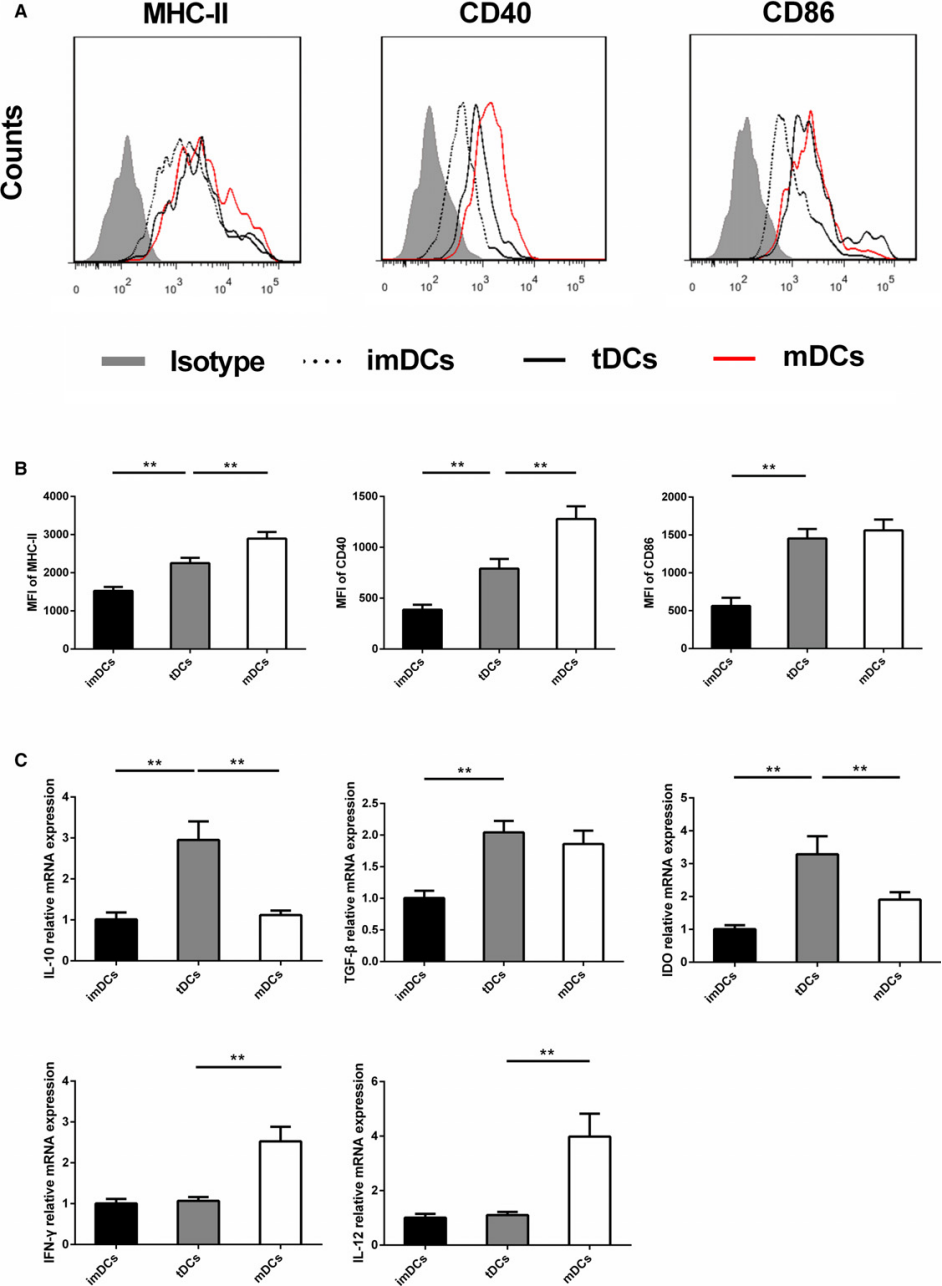
Interleukin (IL)–37 plus troponin I (TnI)–treated dendritic cells (DCs) display tolerogenic properties. A, Bone marrow–derived DCs (2×10^5^ cells/well) were cultured in the absence of stimulus (immature DCs [imDCs]) or in the presence of 1 μg/mL lipopolysaccharide (LPS) (mature DCs [mDCs]) or 10 ng/mL LPS, 30 ng/mL IL‐37, and 1 μg/mL TnIk (tolerogenic DCs [tDCs]). DCs were stained with isotype control antibodies or with specific antibodies against major histocompatibility complex class II (MHC‐II), CD40, and CD86 and analyzed by fluorescence‐activated cell sorting. B, Mean fluorescence intensities (MFIs) for MHC‐II, CD40, and CD86 were quantified. C, Analysis of the mRNA levels of IL‐10, transforming growth factor‐β (TGF‐β), indolamine 2, 3‐dioxygenase (IDO), interferon‐γ (IFN‐γ), and IL‐12 in different DCs groups. n=6 per group. **P<0.01.

We first investigated the role of IL‐37 on the phenotype of BMDCs generated with GM‐CSF and IL‐4. As expected, untreated BMDCs expressed low levels of major histocompatibility complex class II (MHC‐II), CD40, and CD86, a characteristic of imDCs (Figure 6A), and further stimulation with LPS increased levels of MHC‐II, CD40, and CD86 (Figure 6A), a characteristic of mDCs. Compared with mDCs, IL‐37 plus TnI–treated DCs showed lower mean fluorescence intensity (MFI) of MHC‐II molecules and CD40 (Figure 6A and 6B). However, level of CD86 was comparable between DCs loaded with IL‐37 plus TnI and mDCs. As shown in Figure 6C, results showed that DCs loaded with IL‐37 and TnI produced lower mRNA levels of IFN‐γ and IL‐12, as well as higher mRNA levels of IL‐10 and IDO, than the mDC subset. But the mRNA expression of TGF‐β was similar between these two groups. Together, these results indicate that IL‐37 plus TnI–treated DCs acquire a tolerogenic phenotype.

We next quantified MFI of DC‐related cell surface markers of DCs that had been pulsed with either (1) medium, (2) TnI alone (antigen‐loaded DCs), (3) IL‐37 plus TnI (antigen‐loaded tolerogenic DCs), or (4) IL‐37 alone (unloaded tolerogenic DCs). Our data revealed that DCs pulsed with IL‐37 and TnI showed reduced MFI of MHC‐II and CD40 than TnI‐loaded DCs and IL‐37–treated DCs (Figure S4A). In addition, RT‐PCR analysis suggested that the expressions of IL‐10 and IDO were enhanced in the IL‐37– and TnI‐treated group compared with the TnI‐loaded group and the IL‐37–treated group (Figure S4B). Because of the more tolerogenic characteristic of DCs pulsed with IL‐37 and TnI, this DC group was used as tDC in subsequent experiments.

### tDCs Induce Tolerogenic Conditions When Co‐Cultured With CD4^+^T Cells

**Figure 7:**
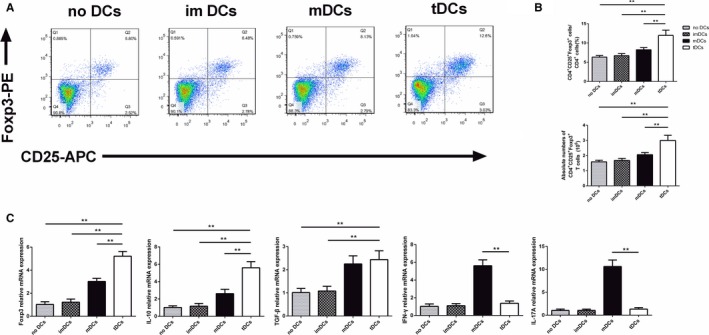
Tolerogenic dentritic cells (tDCs) augment the percentage of regulatory T cells (Tregs) in vitro. A, Splenic CD4^+^ T cells (1×10^6^ cells/mL) were cultured for 3 days in the presence of medium alone or combined with immature DCs (imDCs), mature DCs (mDCs), or tDCs (2×10^5^ cells/mL). The cells were labeled 72 hours later with anti‐CD4, anti‐CD25, and anti‐Foxp3 and analyzed by fluorescence‐activated cell sorting (FACS). FACS data are representative of 1 of 6 to 8 independent experiments (each co‐culture preparation was prepared from a different mouse). B, Graphs represent the average percentages of Tregs (upper) and absolute numbers of Tregs (lower) in CD4^+^ splenic T cells of different groups. C, Analysis of the mRNA levels of Foxp3, interleukin (IL)–10, transforming growth factor‐β (TGF‐β), interferon‐γ (IFN‐γ), and IL‐17A in different groups. No DCs group n=6, imDCs group n=8, mDCs group n=8, and tDCs group n=8. **P<0.01.

To investigate the effect of tDCs on Treg cells expansion, several functional co‐culture experiments were performed. As shown in Figure 7, tDCs markedly increased the CD4^+^CD25^+^Foxp3^+^ Treg cells population and Foxp3 mRNA expression, as measured by both flow cytometry and RT‐PCR, compared with imDCs and mDCs. PBS‐treated, IL‐37–treated, and IL‐37+TnI–treated T cells were also compared. As expected, both the proportion and absolute numbers of CD4^+^CD25^+^Foxp3^+^ Tregs were similar among the above 3 groups (data not shown). Additionally, compared with the mDC subset, tDCs inhibited the mRNA levels of IFN‐γ and IL‐17A (Figure 7B). In contrast, compared with imDCs and mDCs, the tDCs group showed enhanced expression of IL‐10 mRNA (Figure 7B). However, the mRNA level of TGF‐β was comparable between tDCs and mDCs. Taken together, these results imply that the tDCs have a potential to induce tolerance when co‐cultured with CD4^+^T cells.

### tDCs Increase the Number of Tregs and Decrease Th1 and Th17 Cells In Vivo

**Figure 8:**
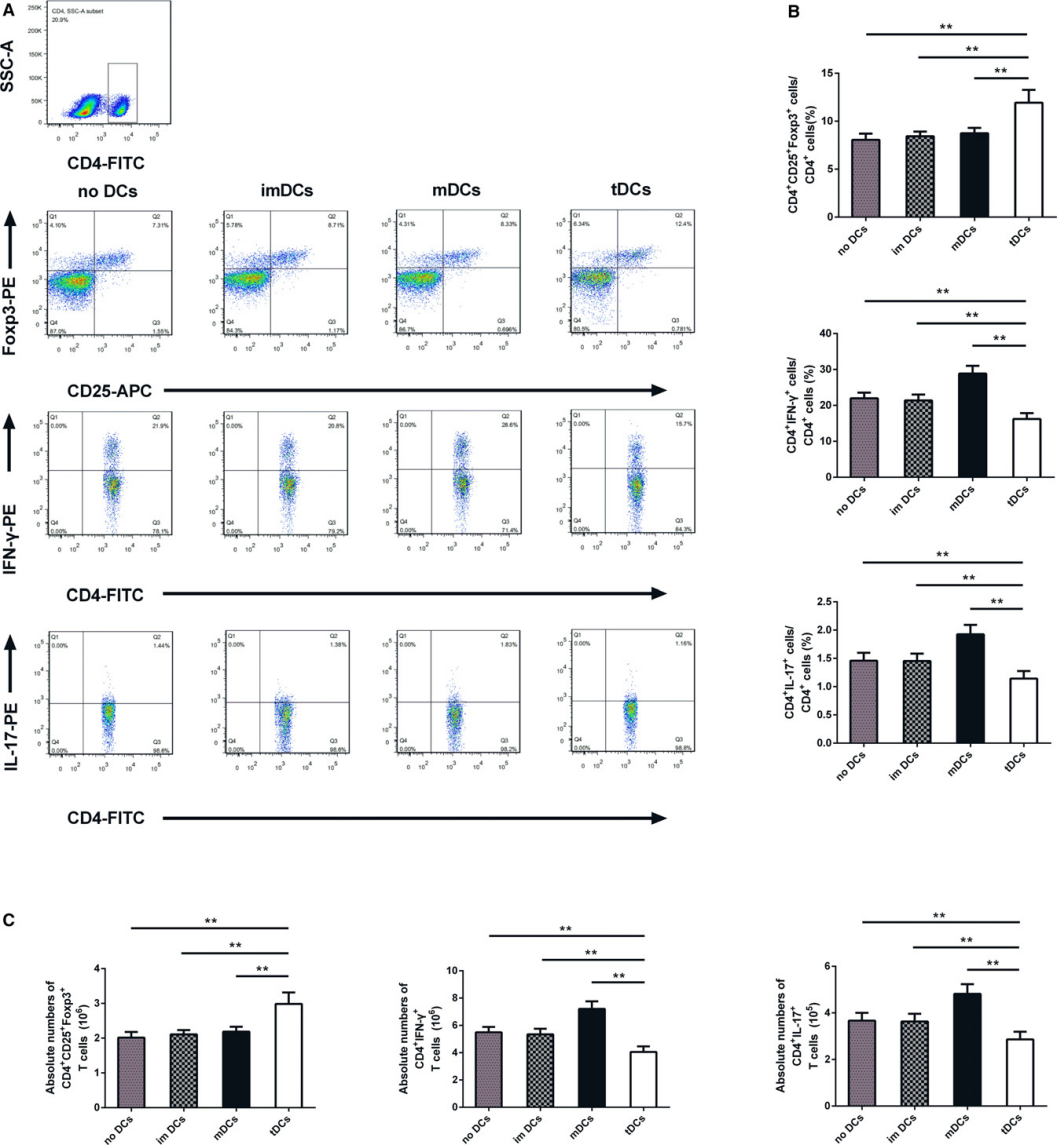
Tolerogenic dentritic cells (tDCs) increase the number of regulatory T cells (Tregs) and decreases T helper (Th)1 and Th17 cells in spleen on day 7 after myocardial infarction (MI). A, CD4^+^ T‐cell subsets were gated, and representative images of Tregs, Th1, and Th17 cells are shown. B, Results of statistical analysis of average percentages of Tregs, Th1, and Th17 cells. C, Absolute numbers of Tregs, Th1, and Th17 cells were counted in the spleen. n=6 per group. **P<0.01.

The findings that IL‐37 plus TnI instructed DCs to acquire tolerogenic properties in vitro prompted us to investigate the influence of tDCs on the development of Tregs, Th1, and Th17 cells in post‐MI mice. As expected, the number of CD4^+^CD25^+^Foxp3^+^ Treg cells significantly increased in the spleens of mice vaccinated with 1×10^6^ tDCs compared with those observed in other groups (Figure 8A and 8B). In contrast, administration of tDCs dramatically decreased the percentage of CD4^+^IFN‐γ^+^ T cells and CD4^+^IL‐17^+^ T cells among the 4 groups (Figure 8A and 8B). Similar results were found for absolute numbers of CD4^+^CD25^+^Foxp3^+^ Tregs, CD4^+^IFN‐γ^+^ T cells, and CD4^+^IL‐17^+^ T cells (Figure 8C). These results were similar with the data in the IL‐37–treated group (Figure 5).

We next measured a series of related mRNA levels in the hearts and spleens, respectively. In the post‐MI hearts, the relative mRNA levels of IFN‐γ, IL‐17A, and IL‐12 were inhibited by tDCs, while the expressions of Foxp3 and IL‐10 were upregulated after administration of tDCs (Figure S5A). However, the mRNA expression of TGF‐β was comparable in the 4 groups (Figure S5A). Furthermore, similar results were discovered in the post‐MI spleens (Figure S5B).

### TnI‐Pulsed tDCs Induced an Antigen‐Specific Treg Cell Population

**Figure 9:**
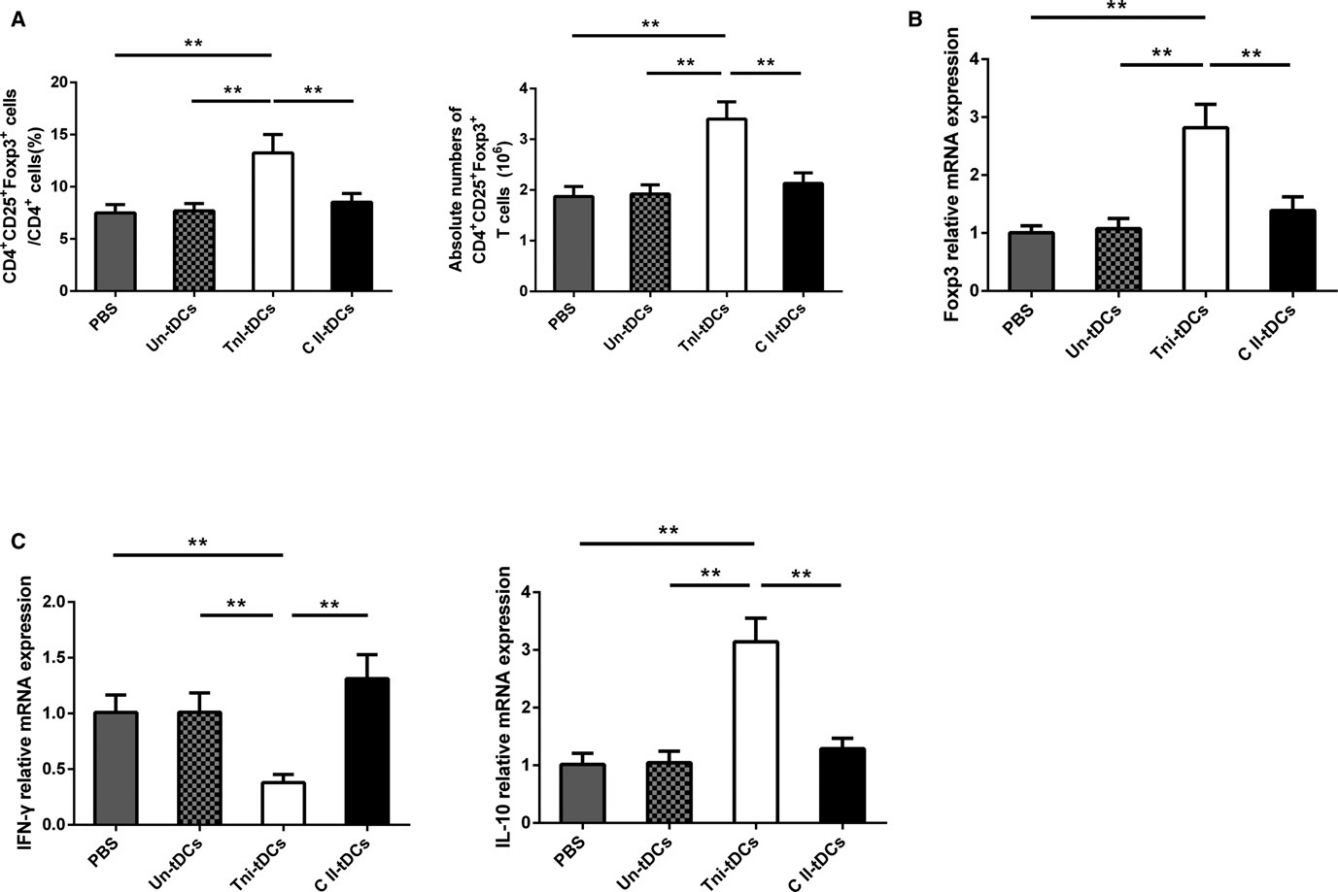
Troponin I (TnI)–pulsed tolerogenic dentritic cells (tDCs) induced an antigen‐specific regulatory T (Treg) cell population. A, Mice received one intravenous injection of PBS, antigen‐unpulsed tolerogenic DCs (Un‐tDCs), TnI‐tDCs, or type II collagen tDCs (CII‐tDCs) (antigen‐mismatched tDCs) 24 hours after myocardial infarction (MI). Six days later, mice were sacrificed and splenocytes harvested. For Treg cell analysis by flow cytometry, the splenocytes were stained with anti‐CD4, anti‐CD25 and then anti‐Foxp3. The percentage and absolute numbers of CD4^+^ CD25^+^Foxp3^+^ cells are shown. B, Foxp3 mRNA levels from the splenocytes (cultured with TnI for 72 hours) were determined by real‐time polymerase chain reaction. C, Analysis of the mRNA levels of interferon‐γ (IFN‐γ) and interleukin (IL)–10 in different groups. n=6 per group. **P<0.01.

To confirm the TnI‐specific Treg cell expansion in vivo, PBS, antigen‐unpulsed, antigen‐mismatched (type II collagen‐pulsed), or TnI‐pulsed tDCs were injected into mice 24 hours after MI. On day 7, mice were sacrificed and splenocytes were harvested. The splenocytes were cultured with TnI (1 μg/mL) for 72 hours and the Treg cell population was evaluated by flow cytometry. At the same time, mRNA levels of Foxp3, IFN‐γ, and IL‐10 in the splenocytes were measured by RT‐PCR. We found that both the proportion and absolute numbers of CD4^+^CD25^+^Foxp3^+^ Tregs were markedly increased in the post‐MI mice that received TnI‐pulsed tDCs (Figure 9A). In addition, increased mRNA levels of Foxp3 and IL‐10 were detected in the splenocytes (cultured with TnI for 72 hours) from post‐MI mice that received TnI‐pulsed tDCs (Figure 9B and 9C). In contrast, decreased IFN‐γ mRNA level was observed in the splenocytes from post‐MI mice injected with TnI‐pulsed tDCs compared with other groups (Figure 9C). These results show that TnI‐loaded tDCs expanded the population of TnI‐specific Treg cells, demonstrating that the Treg cells were antigen specific.

### tDCs Prevent Ventricular Remodeling After MI

**Figure 10:**
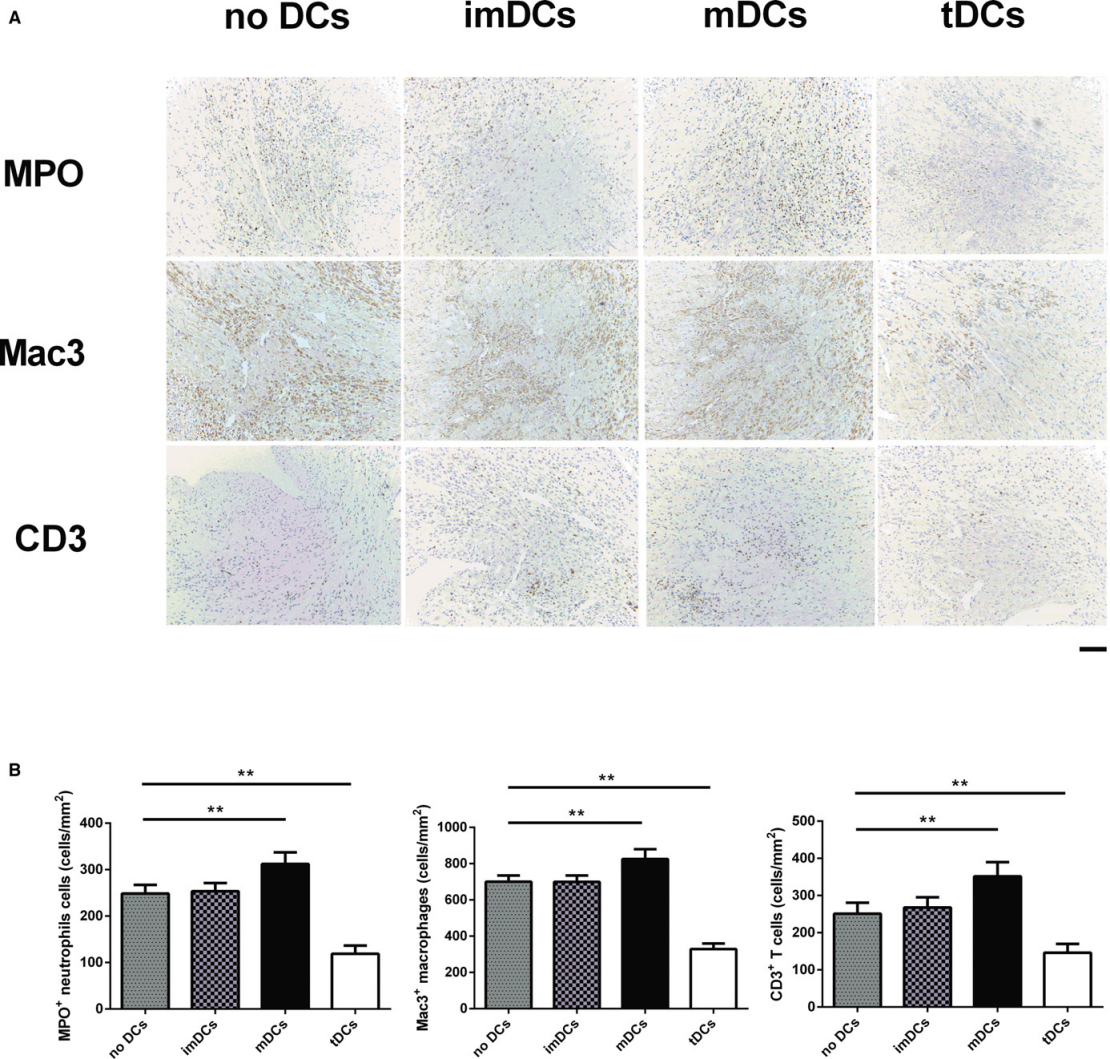
Tolerogenic dentritic cells (tDCs) decrease inflammatory cells infiltration in the infarcted heart. A, Representative images of infiltration of myeloperoxidase (MPO^+^) neutrophils, mouse CD107b (Mac3^+^) macrophages, and CD3^+^ T cells in the border area of the infarct hearts. Images for neutrophils and macrophages are from day 3 after myocardial infarction (MI), and images for T cells are from day 7 post‐MI. B, Infiltration of neutrophils, macrophages, and T cells were compared between the different groups. n=5 per group. Scale bar: 100 μm. **P<0.01.

The in vivo and in vitro studies described above led us to assess the capacity of tDCs to prevent ventricular remodeling after MI. MI mice received a single injection of no DCs, imDCs, tDCs, or mDCs. Immunohistochemical analysis showed that tDCs ameliorated MPO^+^ neutrophils, Mac3^+^ macrophages, and CD3^+^ T‐cells infiltration in the post‐MI hearts compared with no DCs, whereas mDCs deteriorated infiltration of these inflammatory cells into the infarcted myocardium (Figure 10). The data imply that tDCs inhibit inflammatory response in the infarcted heart.

**Figure 11:**
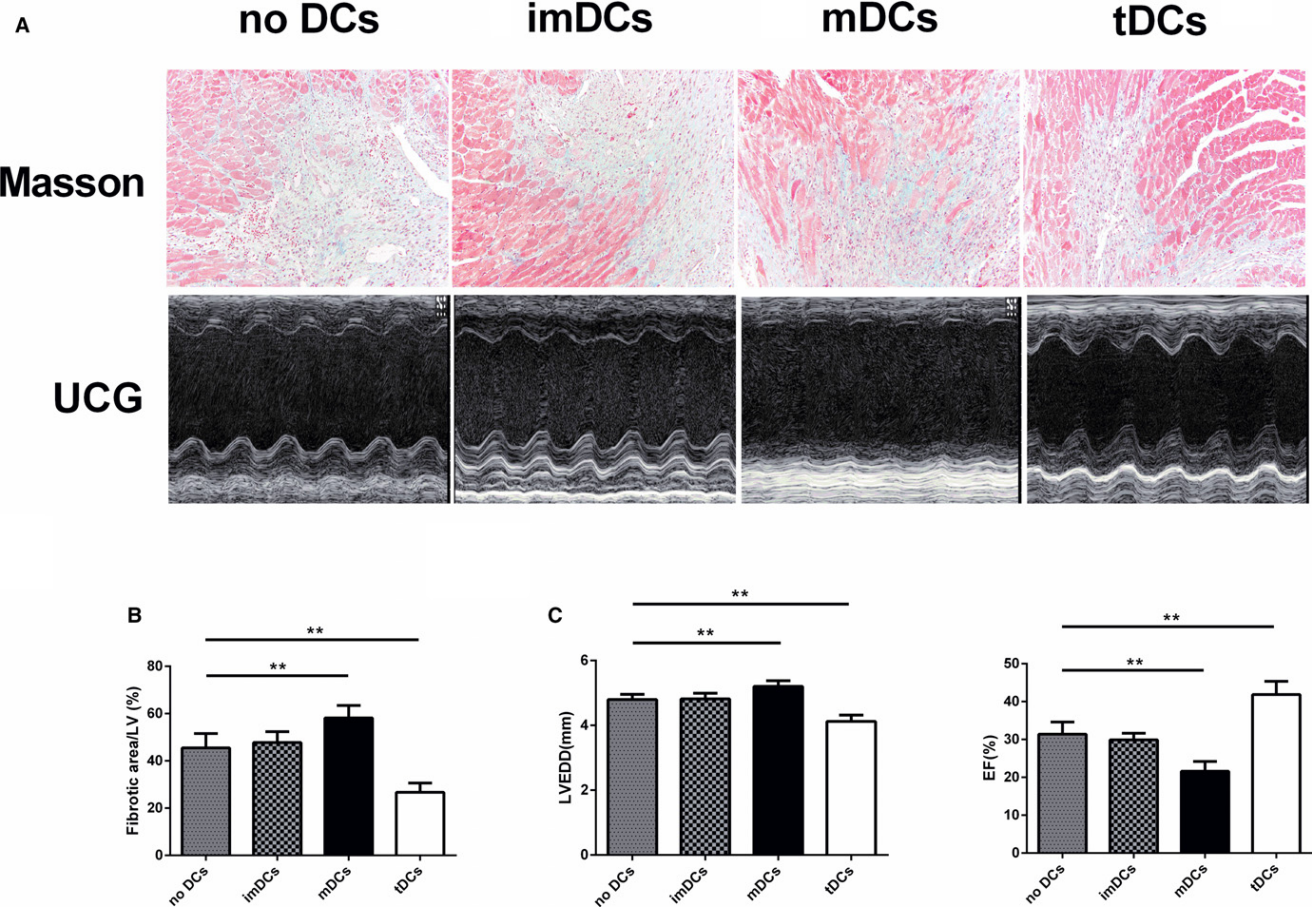
Adoptive transfer of tolerogenic dendritic cells (tDCs) inhibited cardiac fibrosis and prevented left ventricular function following myocardial infarction (MI). A, Representative Masson's trichrome staining images of collagen deposition (blue) in the border area of the infarct hearts on day 28 post‐MI (upper), and representative M‐mode echocardiography images of the left ventricle (LV) on day 28 post‐MI (lower). B, The extent of fibrosis, as assessed by the fibrotic area/LV, was compared among the different groups. C, Data for left ventricular end‐diastolic dimension (LVEDD) and ejection fraction (EF%) in the 4 groups of mice. n=6 per group. **P<0.01.

We next investigated the influence of tDCs on myocardial fibrosis and cardiac function. The fibrotic areas were smaller in tDCs‐treated hearts than in the no DCs‐treated hearts (Figure 11A and 11B). Echocardiographic assessment consistently revealed that LVEDD was smaller, and EF% was greater in tDCs‐treated mice than in no DCs‐treated mice on day 28 after AMI (Figure 11A and 11C). In contrast, myocardial fibrosis and cardiac function were more serious in the mDCs‐treated group than in the no DCs‐treated group (Figure 11). Thus, post‐MI ventricular remodeling was significantly prevented by treatment with tDCs.

### The Inflammatory Reactions Inhibited by IL‐37 or tDCs are Independent of Infarct Size Post‐MI

**Figure 12:**
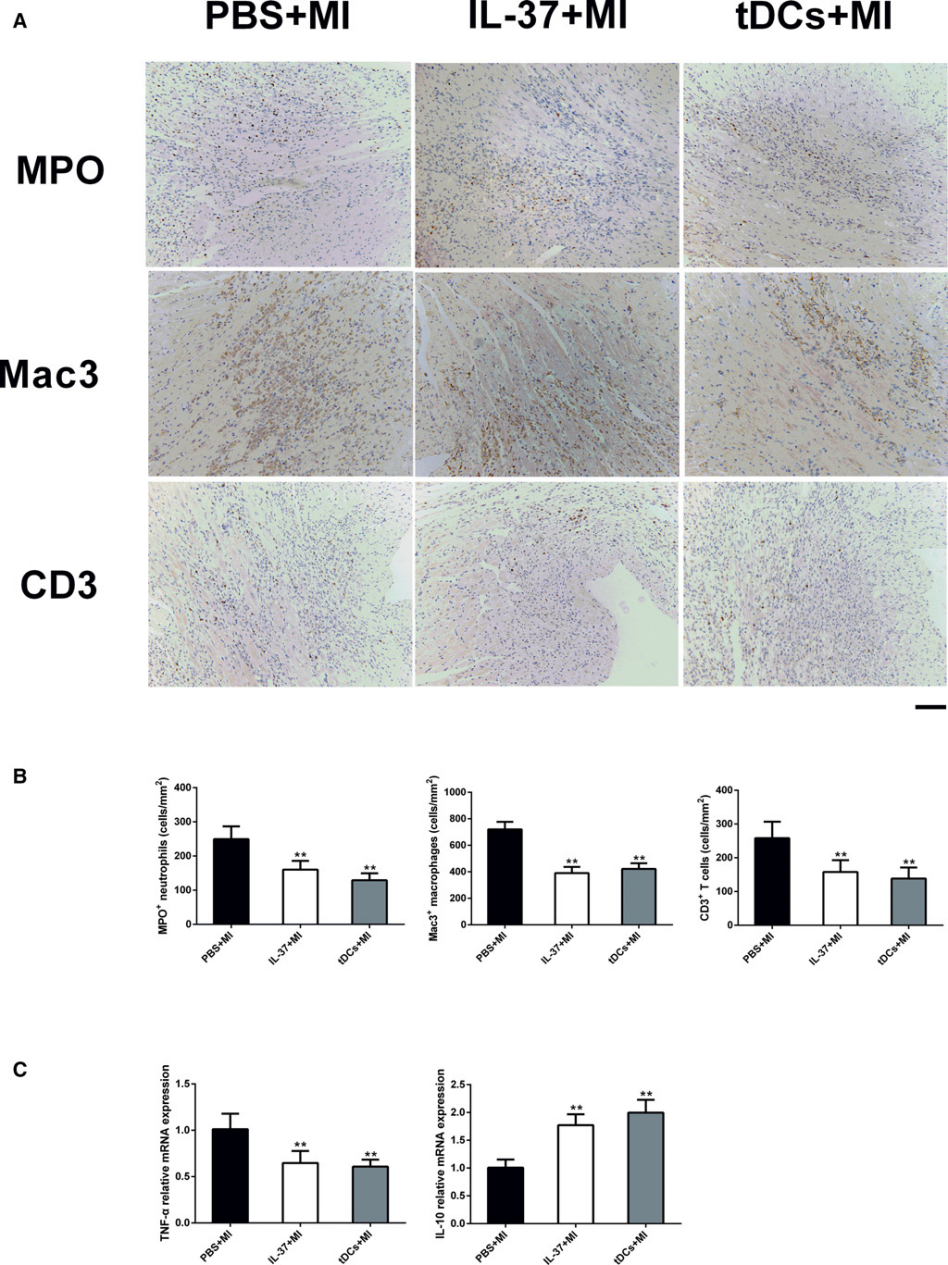
Inflammatory cells infiltration and cytokines expression in the infarcted heart after treatment with interleukin (IL)‐37 or tolerogenic dendritic cells (tDCs) 24 hours post–myocardial infarction (MI). A, Representative images of infiltration of myeloperoxidase (MPO^+^) neutrophils, mouse CD107b (Mac3^+^) macrophages, and CD3^+^ T cells in the border area of the infarct hearts. Images for neutrophils and macrophages are from day 3 after MI, and images for T cells are from day 7 post‐MI. B, Infiltration of neutrophils, macrophages, and T cells were compared between the different groups. C, Analysis of mRNA levels of tumor necrosis factor‐α (TNF‐α) and IL‐10 on day 7 after MI. Data are depicted as fold changes vs PBS+MI. n=5 per group. Scale bar: 100 μm. **P<0.01 vs PBS+MI.

As IL‐37 treatment reduced infarct size and cardiomyocyte death immediately after MI, the secondary immune response may already be altered due to the smaller infarct, rather than IL‐37 treatment. To explore whether the inflammatory response suppressed by IL‐37 or tDCs were independent of infarct size after MI, mice were treated with IL‐37 or tDCs 24 hours post‐MI. As expected, results from immunohistochemical analysis revealed that IL‐37 still ameliorated MPO^+^ neutrophils, Mac3^+^ macrophages, and CD3^+^ T‐cells infiltration in the post‐MI hearts compared with the PBS+MI group (Figure 12A and 12B). In addition, IL‐37 significantly reduced the mRNA level of TNF‐α, whereas the mRNA expression of IL‐10 was markedly upregulated by IL‐37 (Figure 12C). Interestingly, similar results were discovered in the post‐MI hearts after treatment with tDCs (Figure 12). Hence, the inflammatory reactions inhibited by IL‐37 or tDCs are independent of infarct size after MI.

## Discussion

We showed in the present study that treatment with recombinant human IL‐37 significantly ameliorated ventricular remodeling after MI, as demonstrated by smaller infarct size, better cardiac function, lower mortality, restricted inflammatory responses, decreased myocardial fibrosis, and inhibited cardiomyocyte apoptosis. Importantly, we showed for the first time that DCs treated with IL‐37 and TnI displayed a tolerogenic phenotype and that adoptive transfer of these antigen‐loaded tDCs markedly increased the number of Tregs, attenuated the infiltration of inflammatory cells, decreased myocardial fibrosis, and improved cardiac function. Taken together, these data demonstrate that IL‐37 and tDCs treated with IL‐37 plus TnI attenuate cardiac remodeling after MI in mice.

Inflammatory responses and cytokine expression play an important part in the pathogenesis of post‐MI remodeling.^1^, ^2^, ^3^ Strategies that target inflammation after MI require timely resolution of inflammation without interfering with the healing responses.^38^ IL‐37 was regarded as a natural suppressor of both innate and adaptive inflammatory and immune responses.^12^, ^13^ Furthermore, we previously reported that the plasma IL‐37 concentrations are elevated in patients with acute MI.^39^ We therefore hypothesized that IL‐37 may participate in ventricular remodeling after MI as a protective mechanism. In the present study, we observed that administration of recombinant human IL‐37 in mice suppressed infiltration of inflammatory cells (including neutrophils, macrophages, and T lymphocytes) and proinflammatory cytokines (including IL‐6, IL‐1β, and TNF‐α) in the post‐MI hearts, thus ameliorating ventricular remodeling. We also demonstrated that the inflammatory reactions inhibited by IL‐37 are independent of infarct size after MI. Our data were consistent with prior studies showing that restricting the acute inflammatory response attenuated ventricular remodeling.^35^, ^40^, ^41^ Several other studies have revealed similar observations that IL‐37 limited local inflammatory responses by inhibiting recruitment of inflammatory cells.^17^, ^18^, ^42^ Hence, we speculate that downregulation of the accumulation of neutrophils, macrophages, and lymphocytes by IL‐37 may be the potential mechanism for the protective influence of IL‐37 on post‐MI remodeling.

In addition to the early ischemic death of cardiomyocytes, apoptosis accounts for the loss of surviving cardiomyocytes in the late stage of MI.^43^ Several studies have shown that apoptosis is associated with cardiomyocyte death within 4 hours and is also present for up to 30 days after coronary artery ligation.^44^, ^45^ Moreover, inflammation plays an important part in cardiomyocyte apoptosis after MI.^46^, ^47^ In this study, we demonstrated that IL‐37 could attenuate cardiomyocyte apoptosis on days 1 and 28 in the infracted heart and this could be ascribed to the decreased inflammatory response. These results in vivo, along with our previous data from a myocardial ischemia/reperfusion injury model in vitro,^20^ imply that IL‐37 can ameliorate cardiomyocyte apoptosis during both the early and late stages of MI. Several publications reported that antiapoptotic effects on cardiomyocytes could contribute to smaller infarct size.^48^ The influence of IL‐37 on cardiomyocyte apoptosis might explain that administration of IL‐37 could affect the infarct size on days 1 and 28 after MI. Bcl‐2 and in particular the ratio of Bax to Bcl‐2 is a critical determinant of the cell's susceptibility to undergo apoptosis.^49^ We found that Bax/Bcl‐2 ratio was restored in IL‐37–treated mice compared with controls. In addition, the Bax/Bcl‐2 ratio was significantly increased in cardiomyocytes exposed to H_2_O_2_, whereas, when supplemented with IL‐37, decreased the ratio. We, therefore, speculate that IL‐37 inhibits cardiomyocyte apoptosis, at least in part, through downregulation of proapoptotic to antiapoptotic molecule ratio of Bcl‐2 family.

Cardiac TnI has become one of the gold‐standard biomarkers for the acute coronary syndrome, as it indicates myocardial cell damage with high sensitivity and specificity. Once it has entered the systemic circulation after MI, the previously concealed autoantigen induces autoimmune responses of both the cellular and humoral immune system, contributing to myocardial injury. Goser et al^50^ demonstrated that mice immunized with murine cTnI prior to LAD ligation showed greater infarct size, higher inflammation scores, more fibrosis, and reduced cardiac function. Furthermore, Okazaki et al^51^ revealed that autoantibodies against cTnI induce the development of cardiac dysfunction and dilation by stimulating the Ca^2+^ influx in cardiomyocytes. More importantly, one previous publication reported that tolerance induction by nasal vaccination with troponin reduced ischemia/reperfusion injury.^52^ Therefore, tolerance induction based on TnI may provide a novel therapeutic intervention for treatment of ventricular remodeling after MI.

DCs have the ability to induce both immunity and tolerance. Semimature DCs are tolerogenic, whereas fully mature DCs are immunogenic.^24^ In contrast to immunogenic mDCs, it has been confirmed that the effects of such tDCs are mediated by reduced levels of costimulatory molecules and MHC‐II, lower expression of IL‐12, and more secretion of immunosuppressive cytokines such as IL‐10, thereby inducing upregulation of Treg cells and delivering inadequate signals for effector T‐cells activation.^25^, ^53^, ^54^, ^55^ Importantly, Nold et al^12^ and Luo et al^13^ revealed that both BMDCs and splenic DCs of IL‐37tg displayed reduced expression of costimulatory molecules and MHC‐II after LPS treatment. At the same time, Hermansson et al^56^ showed that immunotherapy with DCs treated with ApoB100 and IL‐10 attenuates atherosclerosis in hypercholesterolemic mice. In the present study, we showed that IL‐37 plus TnI–treated DCs showed decreased MFI of MHC‐II molecules and CD40, lower mRNA levels of IFN‐γ and IL‐12, and also higher mRNA levels of IL‐10 and IDO than the mDCs. These results indicate that DCs treated with IL‐37 and TnI generate a tolerogenic phenotype.

Similar to IL‐37 treatment, administration of a single injection of IL‐37 plus TnI–treated tDCs in post‐MI mice significantly reduced the infiltration of inflammatory cells, ameliorated myocardial fibrosis, and improved cardiac function. We also found that the inflammatory reactions inhibited by tDCs are independent of infarct size after MI. However, adoptive transfer of mDCs led to increased inflammatory cells infiltration, aggravated myocardial fibrosis, and exacerbated cardiac function. The present data indicate that proper modification of DCs could be a useful strategy for the treatment of post‐MI remodeling. It has been demonstrated that insufficient recruitment of Treg cells results in exacerbating ventricular remodeling and Treg cells attenuate cardiac remodeling after MI.^35^, ^57^ In the present study, Foxp3^+^ Treg cells and the expression of Foxp3 mRNA were both increased in CD4^+^ T cells co‐cultured with tDCs, and both a significant suppression of Th1 and Th17 cells immunity and marked expansion of Treg cells were observed in mice adoptive transferred with such DCs. Furthermore, our results also showed that these TnI‐loaded tDCs expanded the population of TnI‐specific Treg cells, demonstrating that the Treg cells were antigen specific. These results point to inhibition of cell‐mediated immune responses as a likely mechanism. This is consistent with one previous study that IL‐37–induced tDCs impaired their role in prime T cells and promoted their potential to induce Treg cells.^13^ In addition, IL‐12 is one of the most important cytokines for differentiation of naive T cells into effector Th1 cells, with following production of IFN‐γ.^58^ This further validates the present data that the influence of tDCs on post‐MI remodeling is mediated by inhibition of effector T‐cell immunity.

It is well accepted that IL‐10 is one of the most potent anti‐inflammatory cytokines of Treg cells. In the present study, we found that the level of IL‐10 was increased after treatment with IL‐37 or adoptive transfer with tDCs. One possible explanation is that direct secretion by tDCs and/or expansion of Treg cells are the source of elevated IL‐10. Several previous studies investigated the effect of IL‐10 in ventricular remodeling post‐MI. Frangogiannis et al^59^ reported that IL‐10 knockout mice displayed similar ventricular remodeling with wild‐type mice after MI. However, Dimmeler et al^60^ and Kishore et al^61^ showed that exogenous administration of IL‐10 attenuated ventricular remodeling. Taken together, these studies reveal that endogenous absence of IL‐10 is not adequate to influence ventricular remodeling after MI, whereas exogenous treatment exhibits a protective role. This is in line with our findings that the induction of IL‐10 by IL‐37 or IL‐37 plus TnI–treated tDCs appears to be a unique component of its beneficial effects in the infarcted heart. Nevertheless, TGF‐β, another effector cytokine of Treg cells, was comparable after administration with IL‐37 or adoptive transfer with tDCs, indicating that this anti‐inflammatory cytokine may be not associated with the protection of IL‐37 or tDCs in post‐MI remodeling. TGF‐β is produced by many cell types and its complicated regulation may be the reason for this observation.^62^

So far, the receptors for IL‐37 have not been fully investigated. As shown by a receptor pull‐down assay, IL‐37 has been demonstrated to bind to IL‐18Rα.^63^ However, the affinity of IL‐37 for IL‐18Rα is much lower than that of IL‐18.^64^ IL‐37 does not act as a receptor antagonist for IL‐18 activity, such as induction of IFN‐γ.^15^ However, IL‐37 binds to IL‐18–binding protein (IL‐18BP), which is the natural antagonist of IL‐18.^15^, ^65^ Complex formation between IL‐37 and IL‐18BP may be responsible for the finding that IL‐37 reduces the IL‐18 activity in the presence of low concentrations of IL‐18BP.^15^ Recently, two studies have demonstrated that the multifaceted anti‐inflammatory activity of IL‐37 requires the receptors IL‐18Rα and TIR‐8 (SIGIRR).^66^, ^67^ IL‐18Rα and TIR‐8 /SIGIRR knockout mice are required to further investigate the molecular mechanisms of this interaction in post‐MI remodeling. However, another 2 limitations of the present study should be considered. On the one hand, the number of our samples is relatively small. On the other hand, we have revealed 3 independent mechanisms including the regulation of inflammatory responses, inhibition of myocardial fibrosis, and suppression of cardiomyocyte apoptosis. According to the present results, it is difficult to distinguish which one is the potential mechanism for the beneficial effect of IL‐37. Further studies are needed to address this issue.

## Conclusions

The present study demonstrated that DCs treated with IL‐37 and TnI display a tolerogenic phenotype and that IL‐37 and IL‐37–treated TnI‐loaded tDCs are likely to play an immunoprotective role during the post‐MI remodeling, suggesting that IL‐37 or adoptive transfer of IL‐37 plus TnI–treated tDCs may represent a novel therapeutic strategy for ventricular remodeling after MI.

## Sources of Funding

This work was supported by 3 grants from the National Natural Science Foundations of China (81470420 to Zeng, 81270354 to Zeng, and 81300213 to Zhong).

## Disclosures

None.

## Supporting information


**Table S1.** Methods to Generate Different DC Subsets
**Figure S1.** The ST segment was elevated after permanent ligation of the LAD. LAD indicates left anterior descending artery.
**Figure S2.** Expression levels of inflammatory cytokines in the infarcted heart. A, Representative images of Western blot and quantitative analysis of these proinflammatory cytokines in heart tissue on day 7 post‐MI. B, Representative images of Western blot and quantitative analysis of anti‐inflammatory cytokines in heart tissue on day 7 post‐MI. Data are depicted as fold changes vs sham. n=6 per group.**P<0.01 vs sham and ^##^ *P*<0.01 vs PBS+MI. MI indicates myocardial infarction.
**Figure S3.** IL‐37 inhibits oxidative stress‐induced cardiomyocyte apoptosis via restoration of the Bax/Bcl‐2 ratio. A, Real‐time PCR determined mRNA level of Bax and Bcl‐2 in the infarcted heart on day 1 after MI. The results were also expressed as ratio of Bax/Bcl‐2. B, Real‐time PCR determined mouse neonatal cardiomyocyte Bcl‐2 and Bax mRNA levels. The results were expressed as Bax/Bcl‐2 ratio. n=6 per group. **P<0.01 vs sham and ^##^ *P*<0.01 vs PBS+MI. IL indicates interleukin; MI, myocardial infarction; PCR, polymerase chain reaction.
**Figure S4.** IL‐37 plus TnI–treated DCs exhibit more tolerogenic properties. A, BMDCs (2×10^5^ cells/well) were cultured in the absence of stimulus (imDCs) or in the presence of 10 ng/mL LPS and 1 μg/mL TnI (antigen‐loaded DCs) or 10 ng/mL LPS, 30 ng/mL IL‐37 and 1 μg/mL TnI (antigen‐loaded tolerogenic DCs) or 10 ng/mL LPS and 30 ng/mL IL‐37 (unloaded tolerogenic DCs) for 4 hours. DCs were stained with isotype control antibodies or with specific antibodies against MHC‐II, CD40, and CD86 and analyzed by FACS. MFIs for MHC‐II, CD40, and CD86 were quantified. B, Analysis of the mRNA levels of IL‐12, IL‐10, and IDO in different DCs groups. n=6 per group. *P<0.05 and **P<0.01. BMDC indicates bone marrow–derived dendritic cells; DCs, dendritic cells; FACS, fluorescence‐activated cell sorting; IDO, indolamine 2, 3‐dioxygenase; IL, interleukin; LPS, lipopolysaccharide; MFI, mean fluorescence intensity; TnI, troponin I.
**Figure S5.** Function of tDCs on cytokines expression in the infarct heart and spleen. A, Analysis of mRNA levels of IFN‐γ, IL‐17A, IL‐12, Foxp3, IL‐10, and TGF‐β in the infarct heart on day 7 after MI. B, Analysis of these mRNA levels in the spleen on day 7 after MI. n=6 per group. **P<0.01. IFN‐γ indicates interferon‐γ; IL, interleukin; MI, myocardial infarction; mRNA, messenger ribonucleic acid; tDCs, tolerogenic DCs; TGF‐β, transforming growth factor‐β.
