# Supplementary material for: Interleukin‐37 and Dendritic Cells Treated With Interleukin‐37 Plus Troponin I Ameliorate Cardiac Remodeling After Myocardial Infarction
Source: J Am Heart Assoc. 2016 Dec 5;5(12):e004406. doi: 10.1161/JAHA.116.004406 (PMC5210436; doi:10.1161/JAHA.116.004406)
Supplement: Supplementary file 1 — Table S1. Methods to Generate Different DC Subsets Figure S1. The ST segment was elevated after permanent ligation of the LAD. LAD indicates left anterior descending artery. Figure S2. Expression levels of inflammatory cytokines in the infarcted heart. A, Representative images of Western blot and quantitative analysis of these proinflammatory cytokines in heart tissue on day 7 post‐MI. B, Representative images of Western blot and quantitative analysis of anti‐inflammatory cytokines in heart tissue on day 7 post‐MI. Data are depicted as fold changes vs sham. n=6 per group.**P<0.01 vs sham and ## P<0.01 vs PBS+MI. MI indicates myocardial infarction. Figure S3. IL‐37 inhibits oxidative stress‐induced cardiomyocyte apoptosis via restoration of the Bax/Bcl‐2 ratio. A, Real‐time PCR determined mRNA level of Bax and Bcl‐2 in the infarcted heart on day 1 after MI. The results were also expressed as ratio of Bax/Bcl‐2. B, Real‐time PCR determined mouse neonatal cardiomyocyte Bcl‐2 and Bax mRNA levels. The results were expressed as Bax/Bcl‐2 ratio. n=6 per group. **P<0.01 vs sham and ## P<0.01 vs PBS+MI. IL indicates interleukin; MI, myocardial infarction; PCR, polymerase chain reaction. Figure S4. IL‐37 plus TnI–treated DCs exhibit more tolerogenic properties. A, BMDCs (2×10^5^ cells/well) were cultured in the absence of stimulus (imDCs) or in the presence of 10 ng/mL LPS and 1 μg/mL TnI (antigen‐loaded DCs) or 10 ng/mL LPS, 30 ng/mL IL‐37 and 1 μg/mL TnI (antigen‐loaded tolerogenic DCs) or 10 ng/mL LPS and 30 ng/mL IL‐37 (unloaded tolerogenic DCs) for 4 hours. DCs were stained with isotype control antibodies or with specific antibodies against MHC‐II, CD40, and CD86 and analyzed by FACS. MFIs for MHC‐II, CD40, and CD86 were quantified. B, Analysis of the mRNA levels of IL‐12, IL‐10, and IDO in different DCs groups. n=6 per group. *P<0.05 and **P<0.01. BMDC indicates bone marrow–derived dendritic cells; DCs, dendritic cells; FACS, fluorescence‐activated cell sorting; IDO, indo [file JAH3-5-e004406-s001.pdf]

SUPPLEMENTAL MATERIAL

Table S1. Methods to generate different DC subsets

DC subsets	Methods
Im DCs	No additional incubation
Un-tDCs	CD11c$^+$ im DCs were incubated with 10 ng/mL LPS + 30 ng/mL IL-37 for 4 hours
TnI-tDCs	CD11c$^+$ im DCs were incubated with 10 ng/mL LPS + 30 ng/mL IL-37 + 1 μg/mL TnI for 4 hours
TnI-DCs	CD11c$^+$ im DCs were incubated with 10 ng/mL LPS + 1 μg/mL TnI for 4 hours
C II-tDCs	CD11c$^+$ im DCs were incubated with 10 ng/mL LPS +30 ng/mL IL-37 + 1 μg/mL type II collagen for 4 hours
MDCs	CD11c$^+$ im DCs were incubated with 1 μg/mL LPS for 24 h

Low dose of LPS (10 ng/mL) was used to start DC maturation. Im DCs indicates immature DCs; Un-tDCs, antigen-unpulsed tolerogenic DCs; TnI-tDCs, TnI-pulsed tolerogenic DCs; TnI-DCs, TnI-pulsed DCs; C II-tDCs, type II collagen-pulsed tolerogenic DCs; MDCs, mature DCs; LPS, lipopolysaccharide; IL, interleukin; TnI, troponin I. LPS and type II collagen were from Sigma-Aldrich, St Louis, MO, USA, recombinant human IL-37 was bought from Adipogen AG, Liestal, Switzerland, and TnI was from Fitzgerald Industries International, Inc.

Figure S1.

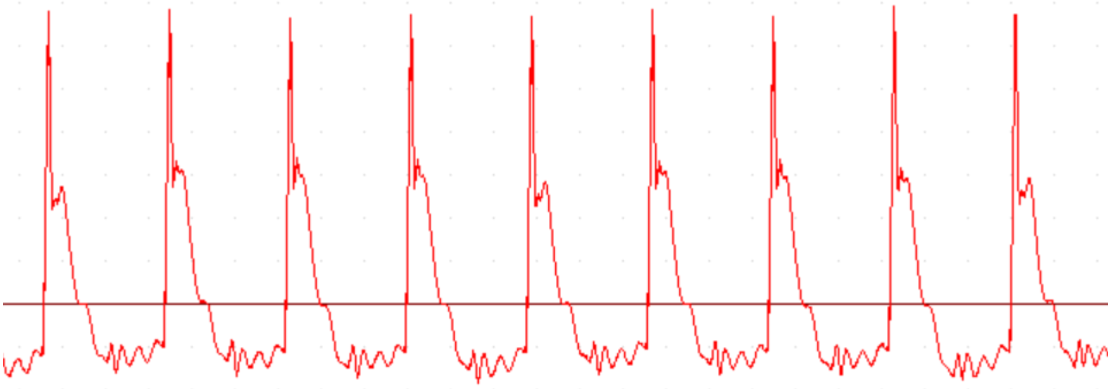

Figure S2.

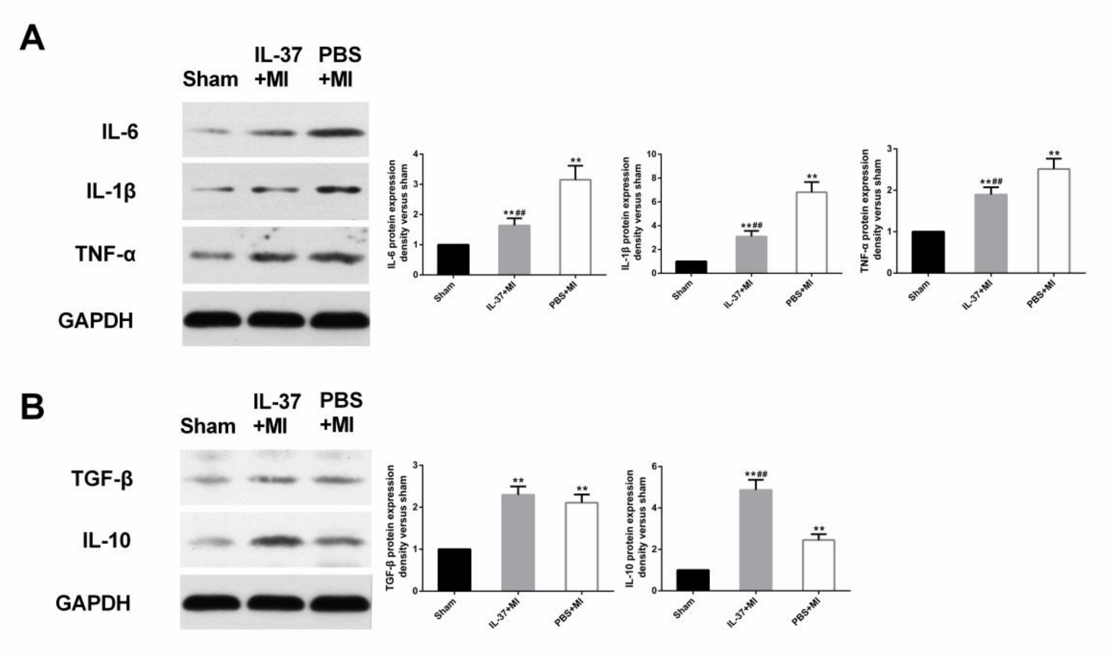

Figure S3.

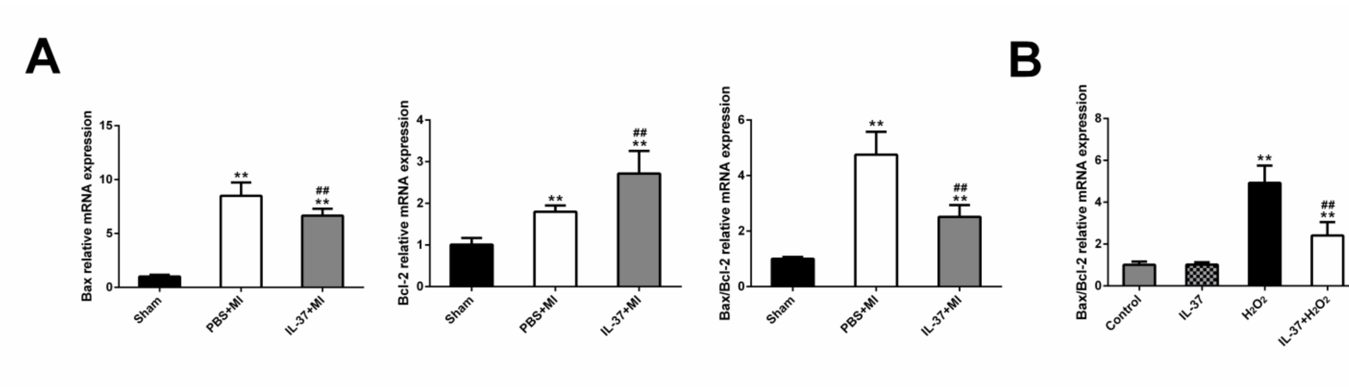

Figure S4.

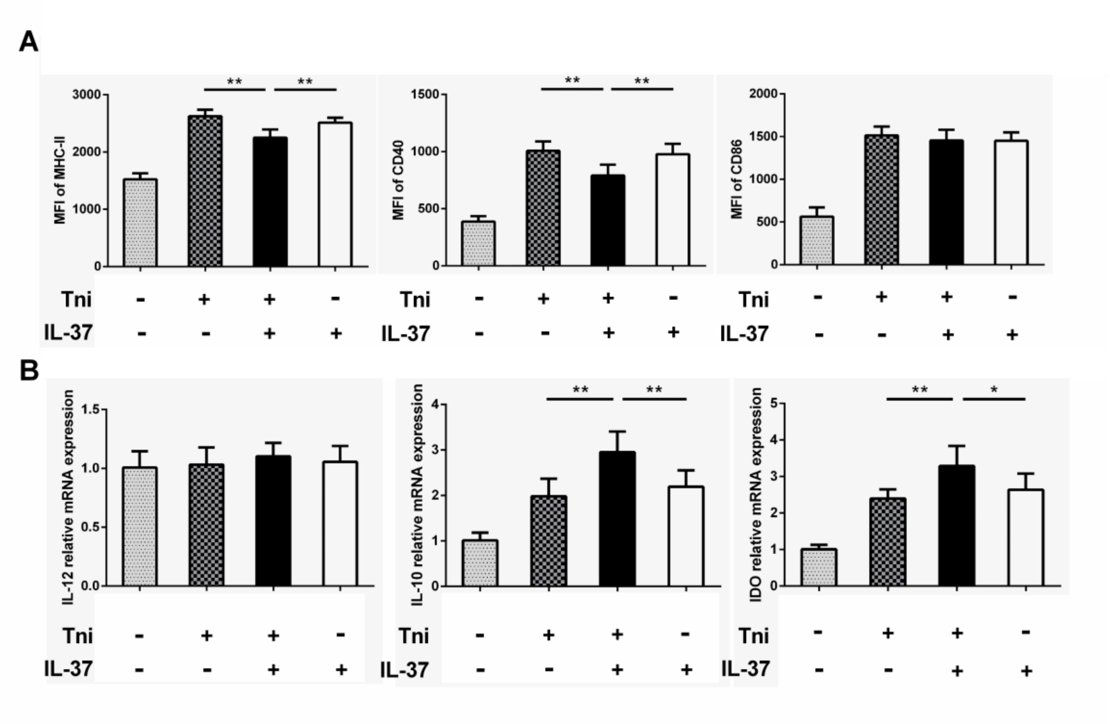

Figure S5.

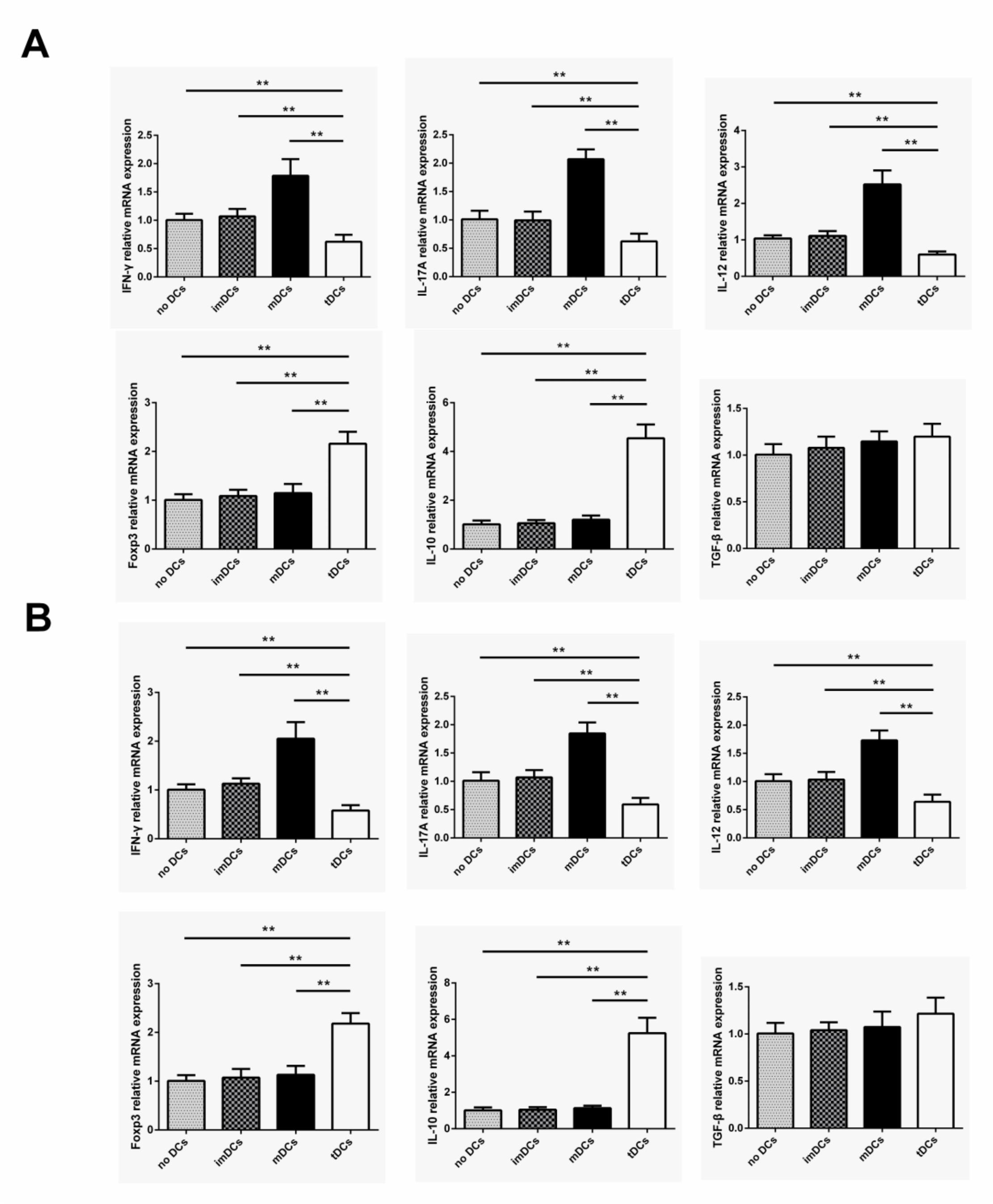

A
B
IFN-γ relative mRNA expression
IL-17A relative mRNA expression
IL-12 relative mRNA expression
Foxp3 relative mRNA expression
IL-10 relative mRNA expression
TGF-β relative mRNA expression
no DCs
imDCs
mDCs
tDCs
**

Supplemental Figure Legends:

Figure S1. The ST-segment was elevated after permanent ligation of the LAD. LAD indicates left anterior descending LAD artery.

Figure S2. Expression levels of inflammatory cytokines in the infarcted heart. A, Representative images of Western blot and quantitative analysis of these pro-inflammatory cytokines in heart tissue on day 7 post-MI. B, Representative images of Western blot and quantitative analysis of anti-inflammatory cytokines in heart tissue on day 7 post-MI. Data are depicted as fold changes versus sham. n = 6 per group. **P<0.01 versus sham and ##P<0.01 versus PBS+MI. MI indicates myocardial infarction.

Figure S3. IL-37 inhibits oxidative stress-induced cardiomyocyte apoptosis via restoration of the Bax/Bcl-2 ratio. A, Real-time PCR determined mRNA level of Bax and Bcl-2 in the infarcted heart on day 1 after MI. The results were also expressed as ratio of Bax/Bcl-2. B, Real-time PCR determined mouse neonatal cardiomyocyte Bcl-2 and Bax mRNA levels. The results were expressed as Bax/Bcl-2 ratio. n = 6 per group. **P<0.01 versus sham and ##P<0.01 versus PBS+MI. PCR indicates polymerase chain reaction; MI, myocardial infarction.

Figure S4. IL-37 plus Tni treated DCs exhibit more tolerogenic properties. A,

BM-DCs (2×10^5 cells/well) were cultured in the absence of stimulus (imDCs) or in the presence of 10 ng/mL LPS and 1 μg/mL Tni (antigen-loaded DCs) or 10 ng/mL LPS, 30 ng/mL IL-37 and 1 μg/mL Tni (antigen-loaded tolerogenic DCs) or 10 ng/mL LPS and 30 ng/mL IL-37 (unloaded tolerogenic DCs) for 4 hours. DCs were stained with isotype control antibodies or with specific antibodies against MHC-II, CD40, and CD86 and analyzed by FACS. MFIs for MHC-II, CD40, and CD86 were quantified. B, Analysis of the mRNA levels of IL-12, IL-10, and IDO in different DCs groups. n = 6 per group. *P<0.05 and **P<0.01. IL indicates interleukin; Tni, troponin I; BM, bone marrow; DCs, dendritic cells; LPS, lipopolysaccharide; FACS, fluorescence-activated cell sorting; MFI, mean fluorescence intensity; IDO, indolamine 2, 3-dioxygenase.

Figure S5. Function of tDCs on cytokines expression in the infarct heart and spleen. A, Analysis of mRNA levels of IFN-γ, IL-17A, IL-12, Foxp3, IL-10 and TGF-β in the infarct heart on day 7 after MI. B, Analysis of these mRNA levels in the spleen on day 7 after MI. n = 6 per group. **P<0.01. TDCs indicate tolerogenic DCs; mRNA, messenger ribonucleic acid; IFN-γ, interferon-γ; IL, interleukin; TGF-β, transforming growth factor-β; MI, myocardial infarction.
